# Mitf Links Neuronal Activity and Long-Term Homeostatic Intrinsic Plasticity

**DOI:** 10.1523/ENEURO.0412-19.2020

**Published:** 2020-04-09

**Authors:** Diahann A. M. Atacho, Hallur Reynisson, Anna Thora Petursdottir, Thor Eysteinsson, Eirikur Steingrimsson, Petur Henry Petersen

**Affiliations:** 1 Department of Anatomy, BioMedical Center, Faculty of Medicine; 2 Department of Physiology, BioMedical Center, Faculty of Medicine; 3 Department of Biochemistry and Molecular Biology, BioMedical Center, Faculty of Medicine; 4 Department of Pharmacology, BioMedical Center, Faculty of Medicine, University of Iceland, Reykjavik 101, Iceland

**Keywords:** genetics, hyperactivity, intrinsic plasticity, Kcnd3, potassium channel, transcription

## Abstract

Neuroplasticity forms the basis for neuronal circuit complexity and differences between otherwise similar circuits. We show that the microphthalmia-associated transcription factor (*Mitf*) plays a central role in intrinsic plasticity of olfactory bulb (OB) projection neurons. Mitral and tufted (M/T) neurons from *Mitf* mutant mice are hyperexcitable, have a reduced A-type potassium current (I_A_) and exhibit reduced expression of *Kcnd3*, which encodes a potassium voltage-gated channel subunit (Kv4.3) important for generating the I_A_. Furthermore, expression of the *Mitf* and *Kcnd3* genes is activity dependent in OB projection neurons and the MITF protein activates expression from *Kcnd3* regulatory elements. Moreover, *Mitf* mutant mice have changes in olfactory habituation and have increased habituation for an odorant following long-term exposure, indicating that regulation of *Kcnd3* is pivotal for long-term olfactory adaptation. Our findings show that *Mitf* acts as a direct regulator of intrinsic homeostatic feedback and links neuronal activity, transcriptional changes and neuronal function.

## Significance Statement

These findings broaden the general understanding of transcriptional regulation of intrinsic plasticity in learning and memory. Regulation of intrinsic plasticity has wide-ranging implications and fundamental importance for neurologic diseases.

## Introduction

Neuronal plasticity is comprised of activity-dependent changes that alter synaptic or intrinsic excitability of neurons (Sweatt, 2016), thus affecting the neuronal response, the gain control relationship between input and output. Ultimately it connects neuronal activity with changes in neuronal behavior (Sweatt, 2016; Yap and Greenberg, 2018). It can also be homeostatic, i.e., neurons with increased activity becoming less sensitive and neurons with lowered activity more sensitive (Lemasson et al., 1993; Davis, 2006; Turrigiano, 2008, 2011). Synaptic plasticity and intrinsic plasticity are thought to work at different time-scales and to interact and complement each other (Kourrich et al., 2015; Lisman et al., 2018). Although synaptic plasticity is fairly well characterized, much less is known about the molecular mechanisms underlying intrinsic plasticity and its transcriptional regulation (Guzman-Karlsson et al., 2014; Hrvatin et al., 2018).

The olfactory system is an attractive system to study plasticity due to its clear flow of information, well-defined neuronal subtypes, ease of activation with odorants and well-defined neuronal circuits. The olfactory bulb (OB) is the first relay of peripheral olfactory information within the CNS. Each OB in the mouse contains around 2000 glomeruli, spherical neuropil structures connecting peripheral olfactory sensory input and cortical structures. In the OB, two types of projection neurons are designated to each glomerulus. They are activated by olfactory sensory neurons (OSNs) and target other CNS regions. These are the mitral cells (MCs; Kikuta et al., 2013; Tan et al., 2010) located in the MC layer (MCL) and the tufted cells located in the external plexiform layer (EPL). These neurons show remarkable capacity in normalizing sensory input, by amplifying low signals and reducing high signals (Roland et al., 2016). Odor exposure also reduces the sensitivity of mitral and tufted cells (M/T; Chaudhury et al., 2010; Kato et al., 2012). In addition, it has been shown that there is high inter-glomerular functional diversity (Angelo et al., 2012), showing that each glomerulus is, to a large degree, regulated independently by its activity. The OB therefore shows clear changes in gain-control and homeostasic plasticity and it has been suggested that intrinsic changes are fundamental to OB sensory adaptation (Tyler et al., 2007).

The transcription factor microphthalmia-associated transcription factor (MITF), best known as a master regulator of melanocytes (Tachibana et al., 1996), is specifically expressed in the M/T neurons of the OB (Ohba et al., 2015). As the M/T neurons are the key output neurons in the OB circuitry, *Mitf* might play a role in olfaction (Ohba et al., 2015). *Mitf* is also expressed in a subset of tufted cells, the external tufted cells (ETCs), which are excitatory and synapse with MC, but do not project out of the OB (Hayar et al., 2004b; De Saint Jan et al., 2009; Whitesell et al., 2013). Here, we show that *Mitf* links neuronal activity and intrinsic activity-dependent changes in the OB by regulating the expression of the potassium channel subunit *Kcnd3*, leading to a homeostatic response in OB’s projection neurons. Therefore, we suggest that *Mitf* plays a key role in olfactory adaptation and intrinsic homeostatic plasticity.

## Materials and Methods

### Animals

All animal procedures were approved by the Committee on Experimental Animals, according to regulation 460/2017 and European Union Directive 2010/63 (license number 2013-03-01). The mice used in the study (*C57BL/6J* and *C57BL/6J-Mitf^mi-vga9/mi-vga9^*) were maintained at the mouse facility at the University of Iceland. Unless otherwise specified, mice were housed two to five per per cage in a temperature-controlled environment (21–22°C). Unless otherwise specified, mice consumed water and food *ad libitum*. Mice of postnatal day P75–P95 of both genders were used for all experiments.

### Electrophysiology

Primary OB neuronal cultures from P0 to P2 mice were obtained as described by (Hilgenberg and Smith, 2007) with slight modifications. Briefly, mice were decapitated and the head submerged in ethanol and then transferred to the head was placed in Ca^2+^ and Mg^2+^-free HBSS (Invitrogen) containing 1% penicillin/streptomycin (Invitrogen) and 1% amphotericin B (Invitrogen) under a dissecting microscope. OBs were dissected from the heads, the meninges removed and the OBs then trypsinized for 7–10 min at 37°C. The cells were washed with 0.05% trypsin-EDTA before incubation in the trypsin solution. The neurons were then gently resuspended in Neurobasal-A Medium (Invitrogen) supplemented with 1% GlutaMAX (Invitrogen), 1% penicillin/streptomycin, 1% amphotericin B, 2% 50× serum-free B27 Supplement (Invitrogen) and 10% fetal bovine serum (FBS). The cells were diluted to a density of 1 × 10^6^ cells ml^−1^ and plated at 1.25 × 10^5^ cells cm^2^ on 0.1 mg/ml poly-D-lysine hydrobromide (Sigma-Aldrich) and 0.075 mg ml^−1^ PureCol Purfied bovine Collagen Solution, type 1 (Advanced BioMatrix) precoated coverslips in Nunclon δ Surface 24-well plates (Thermo Scientific). Neurons were cultured in Neurobasal-A Medium supplemented with 1% GlutaMAX 1% penicillin/streptomycin, 1% Invitrogen amphotericin B, and 2% 50× serum-free B27 Supplement for 2 d, on which they were placed in GlutaMAX-free Medium, which was replenished every 3 d.

The cultured cells were maintained in a solution with NBM-B27, 1% GlutaMAX until the second day *in vitro* (DIV2) and subsequently in NBM-B27 without GlutaMAX. The medium was replaced every 2–3 d. The mean age of cultures at the time of analysis was 12 ± 3 d. The osmolarity of the solutions was measured with an osmometer and the pipette solution was 319 mOsm, while the extracellular Krebs solution was adjusted to 312 mOsm. Isolated cells on coverslips were placed in a recording chamber, and kept at room temperature. The cells were continuously superfused during recordings with an oxygenated extracellular solution. The extracellular mammalian Krebs solution contained the following: 150 mM NaCl, 5 mM KCl, 2.6 mM CaCl_2_, 2 mM MgCl_2_, 10 mM HEPES, and 5 mM glucose, at pH 7.3. The solution was filtered with a 0.2-μm filter before use. Pipettes were pulled out of 1.5-mm O.D. borosilicate glass capillary tubings (Science Products GmbH) with a two-stage Narishige PP83 horizontal puller (Narishige Co), and then fire-polished to ∼1 μm tip diameter in a Narishige MF-83 microforge. Pipette resistances were 2–6 MΩ when placed in the Krebs solution while filled with the internal solution. The internal solution contained the following: 145 mM KCl, 2 mM MgCl_2_, 10 mM EGTA, 10 mM HEPES, and 0.2 mM NaATP, pH 7.3. The reference Ag/AgCl electrode was placed in a compartment separated from the recording chamber, and electrically linked to the superfused compartment containing the cells with a glass tube bridge filled with 2 M KCl/Agar solution. Recordings were made using the whole-cell configuration of the patch-clamp technique (Hamill et al., 1981) on M/T cells with large nuclei, with an Axopatch 1D patch clamp amplifier (Molecular Devices Inc) in voltage or current clamp mode. The mean membrane resistance of wild-type M/T neurons was 104 ± 20.3 MΩ, and 98 ± 17 MΩ (*t*_(25)_ = 0.234, *p* = 0.82, two-tailed test) in *Mitf* mutant neurons. The mean series resistance of wild-type M/T neurons was 13 ± 2.3 MΩ, and 17 ± 1.6 MΩ (*t*_(25)_ = −1.415, *p* = 0.17, two-tailed test) in *Mitf* mutant neurons. The mean cell capacitance of wild-type neurons was 46 ± 7.3 pF and that of *Mitf* mutant neurons was 56 ± 11 pF (*t*_(25)_ = −0.7308, *p* = 0.47, two-tailed *t test*). The mean resting membrane potentials of wild-type M/T neurons were −58 ± 2 mV and of *Mitf* mutant neurons –57 ± 2 mV, which is not signficantly different (*t*_(11)_ = −0.102, *p* = 0.92, two-tailed *t test*). The measured values of all these biophysical parameters of the cultured cells are comparable to previously published values measured from cultured mouse OB M/T cells (Desmaisons et al., 1999; Fadool et al., 2004). The output of the amplifier was low-pass filtered at 2 kHz and digitized at 10 kHz with a Digidata 1440A (Molecular Devices) 16-bit A/D-D/A converter. Acquisition of recordings and generation of voltage or current pulses were performed with the pClamp 10.0 software (Molecular Devices) in conjunction with the Axopatch 1D amplifier. No corrections were made for liquid junction potentials or leak currents. After a break-in under voltage clamp, the cell was clamped at a holding potential of −70 mV, and then a 5-mV hyperpolarizing voltage step of 50 ms in duration was applied to evoke capacitance transients in the whole-cell current, to estimate the series resistance (Rs), input resistance (Ri), membrane resistance (Rm), and capacitance (Cm) of the cell. Subsequently, the cultured cells were then first voltage clamped at a holding potential of –70 mV, and then stepped to –90 mV for 80 ms. Whole-cell outward currents were then evoked by a protocol using successive depolarizing voltage pulses from –90 mV, 300 ms in duration at +5-mV steps. To separate the A-type current from other outward currents another protocol with a series of voltage pulses 300 ms in duration was then applied to the cell, except that before the depolarizing pulses were delivered, the cell was held at –40 mV for 80 ms, after having been kept hyperpolarized for 55 ms at –90 mV. In order to isolate the A-type current from the total outward current, the current traces evoked by the second protocol, which primarily involves delayed rectifier K^+^ currents, were subtracted from the corresponding traces evoked by the first protocol. The resting membrane potential was recorded immediately after break in and turning to whole-cell current clamp mode with current at zero. Spontaneous membrane potential fluctuations and spiking activity of projection neurons were measured in whole-cell current clamp mode, while holding the cell near the resting potential (−65 ± 5 mV). A depolarizing shift in membrane potential larger than 25 mV was defined in the analysis software as a spike, while a shift of 5 mV but <25 mV was defined as a spontaneous EPSP. The time period for analysis of spontaneous spike frequency in each cell was set as 50 s.

### Immunofluorescence

Mice were transcardially perfused (experimental license number 2014-07-02) with 1× PBS (Dulbecco) followed by 4% paraformaldehyde (PFA; Sigma-Aldrich) in 1× PBS. Following dissection, the brain was postfixed for 2 h in 4% PFA at room temperature. The OBs were sectioned at −20°C, in 20-μm thin sections and kept at 4°C until used for immunofluorescence. Sections were placed in a blocking buffer of 0.1% Triton X-114 (Sigma-Aldrich) and 5% normal goat serum (NGS; Invitrogen) in PBS for 1 h at room temperature. Blocking was followed by incubation with primary antibody (diluted 1:500) in blocking buffer overnight at 4°C. The antibodies used were TBR2/Eomes (Abcam, Ab23345, RRID: AB_778267) and tyrosine hydroxylase (TH; Millipore, MAB318, RRID: AB_2313764). Sections were subsequently incubated for 1 h at room temperature in blocking buffer containing the Alexa Fluor goat anti-mouse 488 or Alexa Fluor goat anti-rabbit 546 (Life Technologies) secondary antibodies (each at 1:1000 dilution) and DAPI (1:1000; Sigma-Aldrich). The sections were then washed and subsequently kept at 4°C until imaging at 20× magnification using an Olympus FV10-MCPSU confocal microscope; two to five images were obtained from the medial OB of each sample. Cells were counted manually. Quantification of the samples was performed under blind condition. The average of cells per sample was calculated and plotted using GraphPad Prism 7.

### TUNEL staining

Fixed frozen sections were obtained as described above. For TUNEL staining of the fixed frozen sections, In Situ Cell Death Detection kit, Fluorescein version 17 (Roche) was used. Images were obtained at 20× magnification using Z-stack 3D images. From each sample, two to three images were obtained from the medial OB. Using the Fiji software, images were stacked and positively stained cells were counted manually. The average number of positive cells per image was calculated using GraphPad Prism 7.

### Histologic analysis

Mice were sacrificed by cervical dislocation and the brain was postfixed for 24 h in 4% formaldehyde (Sigma-Aldrich). Following fixation, the OB was embedded in paraffin and sectioned in 4-μm thin sections. The sections were stained for hematoxylin and eosin (H&E).

### Plasmids

The MITF-M cDNA was cloned into the p3xFlag-CMV14 vector using EcoRI and BamHI restriction sites. The four arginines in the basic domain were mutated to alanines by in situ mutagenesis to generate the construct MITF-B4RA (Fock et al., 2019). The human TYR promoter in pGL3 Basic Luciferase Reporter vector was used for co-transfection assays.

MITF binding sites in the intronic region of KCND3 were identified using the ChIPseq data from Laurette et al. (2015; GSM1517751) and Strub et al. (2011; GSE64137). The data were imported into IGV Tools and binding peaks near KCND3 identified. Similarly, ChIPseq data for H3K4me1 (GSM2476344) and H3K27ac modifications in melanoma cells (GSM2476350) was analyzed (Fontanals-Cirera et al., 2017). Two MITF peaks termed A and B were identified. In order to clone the KCND3_B region, a 655-bp fragment was amplified from genomic DNA of the human 501mel melanoma cells using forward primer 5′-TTGTGAGAGTAGCAGAGTGCTTTGC-3′ and reverse primer 5′-GAGCAGATTCAGAGATCAGAAATCAATGG-3′; the primers also contained restriction sites for KpnI and XhoI. In order to clone the KCND3-A region, a 469-bp fragment was amplified from genomic DNA of 501mel melanoma cells using forward primer 5′-GCTTCTGGAAGGTGAGAGAAGGA-3′ and reverse primer 5′-AGTGTCCTGATAGCCACATTAGGTC-3′; the primers also contained restriction sites for NheI and XhoI. Both fragments were subsequently cloned into the pGL3 Promoter reporter vector.

### Cell culture

HEK293T and N2A neuronal cells were obtained from the Leibniz Institute DSMZ-Deutsche Sammlung von Mikroorganism und Zellkulturen GmbH. HEK293T cells were cultured in DMEM-Glutamax media (Invitrogen) supplemented with 10% FBS (Invitrogen), N2A cells were cultured in DMEM-Glutamax media supplemented with 10% FBS and 1% Non-Essential Amino Acids (Invitrogen). Cells were cultured in 75-cm^2^ Nunc EasYflask with Nunclon δ surface (Thermo Scientific) in a HERAcell 240i incubator (Thermo Scientific) at 37°C and 5% CO_2_. HEK293Ts were passaged when 90% confluency was reached by gently trypsinizing the cells, resuspending them in media and diluting the cells 1:10 and replating them. N2A cells were passaged when 70% confluency was reached by gently trypsinizing the cells, resuspending them in media and diluting the cells 1:5 and replating them.

### Co-transfection assay

N2A and HEK293T cells were plated in Falcon 96-well cell culture plates at 13,800 and 20,000 cells per well, respectively. After 24 h, the N2A and HEK293T cells were transfected with 0.1 μg of DNA mixed with FuGENE at 7:1 and 2.8:1 FuGENE HD to DNA ratios, respectively. This mixture contained equal amounts of the luciferase reporter plasmid, *Renilla* reporter plasmid and MITF-FlagpCMV, MITF-B4RAFlagpCMV, or FlagpCMV. Each combination was plated and transfected in triplicate. Cells were harvested after 24 h of incubation using Dual-Glo Luciferase Assay System (Promega Corporation) as described and the luciferase and *Renilla* luminescence measured separately using a MODULUS II TURNER instrument (Biosystems). To quantify the normalized luminescence, the luciferase values were divided by the *Renilla* luminescence values recorded for each well. Subsequently an average was obtained for the triplicate. These values were then plotted using GraphPad Prism 7.

### Odor exposure

Mice were placed in empty cages, devoid of bedding, food, and water for 1 h in an odor free room. After 1 h, mice were moved to a different room where an open Eppendorf tube containing 60 μl of amyl acetate (Sigma-Aldrich) was taped inside their cage at nose-level. After 30 min, the mice were placed in a new cage without amyl acetate and moved back to the odor free room. The mice were sacrificed by cervical dislocation at different intervals: before amyl acetate exposure, immediately after exposure and 30, 90, and 210 min after exposure to amyl acetate. The OBs were either flash frozen in liquid nitrogen for quantitative real time PCR or placed in OCT medium (Sakura) in a 15 × 15 × 15-mm Tissue-Tek Cryomold (Sakura) and subsequently flash frozen for sectioning and RNA *in situ* hybridization.

### RNA multiplex fluorescent RNA *in situ* hybridization (mFISH)

Mice were sacrificed by cervical dislocation. The OB was dissected out and placed in OCT compound in a 15 × 15 × 15 mm Tissue-Tek Cryomold and flash frozen with liquid nitrogen. The caudal OB was sequentially sectioned unto slides, with two sections per slide, at a thickness of 20 μm. The slides were stored in a −80°C freezer in an airtight bag until use. The samples were pretreated using the protocol RNAscope Sample Preparation and Pretreatment Guide for Fresh Frozen Tissue (Manual RNAScope assay; Advanced Cell Diagnostics). mFISH was subsequently performed according to the manufacturer’s instruction for the RNAscope Fluorescent Multiplex kit (Advanced Cell Diagnostics) with probes 422501 (*Mitf*), 429641-C2 (*Tbr2/Eomes*), 452598-C3 (*Kcnd3*), and 316921-C2 (*c-Fos*). Sections were kept at 4°C until imaging using Z-stack 3D images with the Olympus FluoView FV10 confocal microscope at 30× magnification. Two images were obtained from the medial OB per sample, one image of the glomerular region of the OB and a separate image of the mitral and granule cell (GC) layer (GCL). Using the Fiji software, fluorescent dots per cell were counted, by encircling the cloud of *Tbr2* dots and using this area to count the fluorescent dots of interest. *Tbr2*+ cells located directly below the glomeruli were considered ETCs. Similarly, *Tbr2*+ cells located in the MCL, and not in the EPL, were considered as MCs. In the cases where *Tbr2* coexpression was not determined, *Mitf* expression, localization, and nuclear morphology were used to determine cell type. Average dots per cell was calculated for each sample and plotted using GraphPad Prism 7.

### Quantitative PCR

Mice were sacrificed by cervical dislocation and OBs flash frozen in liquid nitrogen. The samples were kept at −80°C until RNA was isolated using NucleoSpin RNA (Machery Nagel). In order to generate cDNA, the SuperScript II Reverse Transcriptase kit (Invitrogen) was used, using 1 μg of RNA and oligo(T) primers. Mouse specific exon-spanning primers were designed using Primer-blast. The primers-pairs used were the following: *Gapdh* forward 5′-ATGACATCAAGAAGGTGGTG-3′, reverse 5′-CATACCAGGAAATGAGCTTG-3′; *Actin* forward 5′-CACTGTCGAGTCGCGTCC-3′, reverse 5′-TCATCCATGGCGAACTGGTG-3′; *Mitf* forward 5′-AGCAAGAGCATTGGCTAAAGA-3′, reverse 5′-GCATGTCTGGATCATTTGACT-3′; and *c-Fos* forward 5′-TTTCAACGCCGACTACGAGG-3′, reverse 5′-TCTGCGCAAAAGTCCTGTGT-3′. Quantitative qPCR was performed using Power SYBR Green PCR Master Mix (ThermoFisher Scientific). The 2^-ΔΔ^*^C^*^t^ method was used to normalize expression to *C57BL/6J,* relative to *Gapdh* and *Actin* as described previously (Livak and Schmittgen, 2001).

### Behavior

Before performing any behavior experiments, individual mice were placed in separate cages for at least 24 h. Mice were used for a maximum of two behavioral experiments but only once for the same behavioral test. Each mouse was only used once for each habituation/dishabituation experiment. All behavior experiments were performed at 9 P.M., 1 h after the lights were turned off. All behavior was filmed, and the behavior of interest measured as a function of time and plotted using GraphPad Prism 7.

### Hidden food assay

The food pellets were replaced by sweetened cereal, Cocoa Puffs (Nesquik General Mills) for 12 h. After 12 h, all Cocoa Puffs pellets were removed from the food section of the cage and one Cocoa Puffs pellet was placed into the cage. Following overnight starvation (license number 2016-05-01), a cage was filled with 4 cm of bedding and a Cocoa Puffs pellet was hidden in one corner, under the bedding. The mouse was placed in the posterior section of the cage, opposite to the pellet, and the time it took the mouse to find the pellet was determined. Mice which showed obvious distress (tremors, unsteady gait, or unwillingness to explore) and could not find the pellet in under 180 s were removed from the experiment.

### Avoidance assay

A 41.5 × 24 × 18.5 cm cage was sectioned into three equal sections numbered 1–3 and the bottom covered with 0.5 cm of bedding. Before the experiment, 30 μl of water were placed onto a 5 × 5 cm piece of Whatman paper which was then taped onto section 1, at nose height. The mouse was gently placed in section 3 and allowed to roam for 120 s in the cage. After 120 s, the mouse was removed from the cage and the 5 × 5 cm Whatman paper containing 30 μl of water was replaced with a 5 × 5 cm paper of Whatman paper containing 30 μl of propionic acid (Sigma-Aldrich). The mouse was gently placed in section 3 and allowed to roam for 120 s in the cage. After 120 s, the mouse was removed from the cage. The time the mouse spent in each of the three sections, using the nose as the reference point, was measured for both conditions.

### Habituation-dishabituation

In order to perform the habituation-dishabituation experiment, we used the protocol of Lehmkuhl and associates (Lehmkuhl et al., 2014). Mice were kept in individual cages for 24 h. Food and water were subsequently removed, and the mice were kept in an odor free room for 1 h before the experiment. The mice were habituated for 30 s every 5 min by placing 5 μl of a particular odor in the cage, a total of six times, and subsequently exposed to 5 μl of another odor for 30 s. Time spent sniffing the object, or in the direction of the object was determined. Odorants used were almond, lime (brand name Dr. Oetker) and vanilla extracts (brand name Katla). For long-term exposure to odor, the mice were exposed to vanilla odorant for 2 h before habituation-dishabituation with almond-vanilla odor pairs.

### Statistical analysis

No statistical methods were used to predetermine sample size. Grouped analyses were used for the *in vivo* experiments as at least three mice per genotype were used. Quantitative results were analyzed by one-way or two-way ANOVAs, and two-sided unpaired or paired Student’s *t* tests and χ^2^ tests using GraphPad Prism 7. To obtain *p* values, multiple comparisons for ANOVA tests were performed with Livak’s correction or Dunnett’s correction in cases where there was a comparison to a control time point. All numerical results are presented as mean and SEM unless stated otherwise. Degrees of freedom are indicated between brackets.

## Results

### *Mitf* mutant mice have increased numbers of excitatory neurons

**Figure 1.**
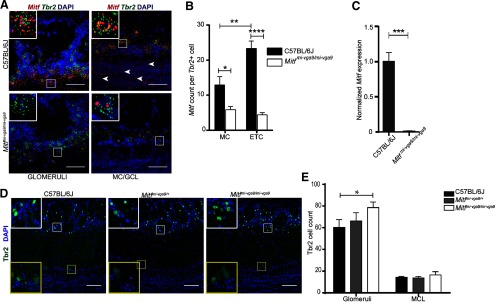
*Mitf* mutant mice have an increase in Tbr+ neurons in the glomerular layer. ***A***, RNA *in situ* hybridization of *Mitf* (red) and *Tbr2* (green) in glomeruli and MC/GCL of wild-type and *Mitf^mi-vga9/mi-vga9^* mice. Scale bars: 50 μm. ***B***, *Mitf* count per *Tbr2*-positive cells below glomeruli and MCL. *N* = 9 per genotype. ***C***, *Mitf* mRNA expression, determined by RT-qPCR. *N* = 6 per genotype. ***D***, Immunofluorescent staining of Tbr2/Eomes (Tbr2+) neurons (green) in the OBs of wild-type, *Mitf^mi-vga9/+^*, and *Mitf^mi-vga9/mi-vga9^* mice. ***E***, Cell count of Tbr2/Eomes (Tbr2+) cells in the glomeruli and MCL of wild-type, *Mitf^mi-vga9/+^*, and *Mitf^mi-vga9/mi-vga9^* mice. *N* = 6 per genotype. The values on the graphs are mean ± SEM DAPI nuclear staining is shown in blue. Scale bars: 100 μm; *p* values were calculated using two-way ANOVA (***B***, ***E***) or two-tailed unpaired Student’s *t* test (***C***); *p < 0.05, **p < 0.01, ***p < 0.001, ****p < 0.0001.

In order to characterize *Mitf* expression in the OB, we used mFISH which generates a single fluorescent dot per transcript detected (Wang et al., 2012), allowing for single-cell analysis of gene expression. Consistent with previous observations (Ohba et al., 2015), this showed that in wild-type mice *Mitf* is expressed in M/T neurons, including in ETCs located in the EPL (Fig. 1*A*,*B*) and at low levels in the GCL (Fig. 1*A*, arrowheads). Interestingly, there is significantly more *Mitf* expressed in the ETCs compared with MCs (*F*_(1,15)_ = 8.94, *p* = 0.0092, two-way ANOVA). Mice homozygous for the *Mitf^mi-vga9^* mutation are white and microphthalmic due to a transgene insertion mutation that affects expression of *Mitf* (Hodgkinson et al., 1993). As expected, *Mitf* expression was decreased in the ETCs (*t*_(14)_ = 7.923, *p* < 0.0001, Sidak multiple comparison test) and MCs (*t*_(14)_ = 2.969, *p* = 0.0202, Sidak multiple comparison) in OBs of *Mitf* mutant mice (Fig. 1*B*,*C*).

**Figure 2.**
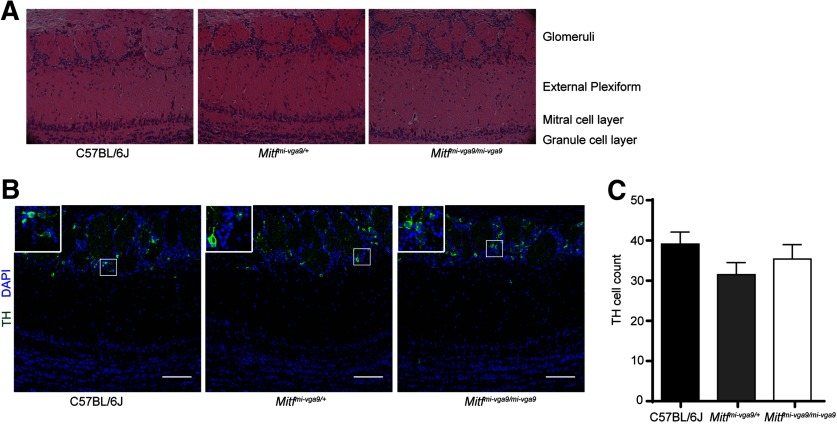
*Mitf^mi-vga9/mi-vga9^* mice have normal OBs. ***A***, Representative images of the H&E histologic analysis of coronal sections of the medial OBs of the indicated genotypes. ***B***, Immunofluorescent staining of TH showing the localization of TH in the glomeruli of the indicated genotypes. ***C***, Cell count of TH+ cells of the OB of wild-type, *Mitf^mi-vga9/+^*, and *Mitf^mi-vga9/mi-vga9^* mice. *N* = 6 per genotype. The values on the graphs are mean ± SEM DAPI nuclear staining is shown in blue. Scale bar: 100 μm; *p* values were calculated using one-way ANOVA (***C***).

**Figure 3.**
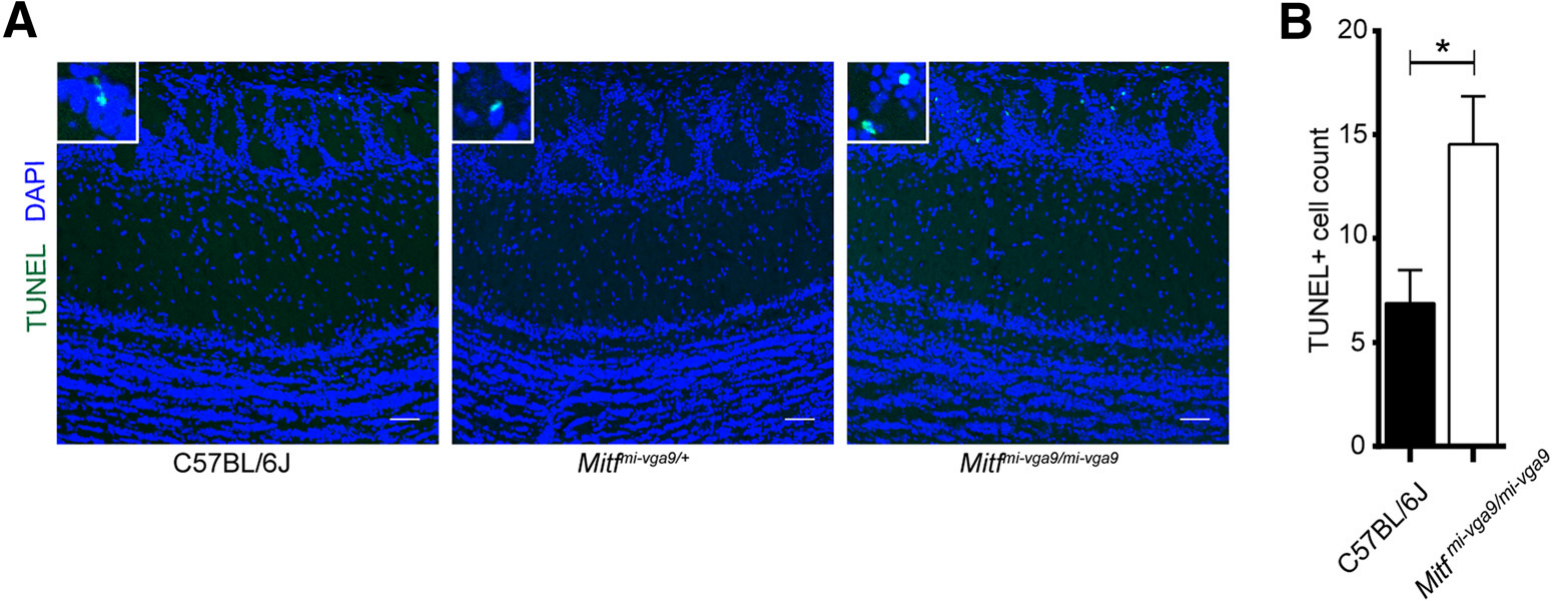
Increase in apoptosis in OBs of *Mitf^mi-vga9/mi-vga9^* mice. ***A***, TUNEL staining of sections of OBs of the indicated genotypes, showing localization of apoptotic cells. ***B***, The number of apoptotic cells of OB of wild-type and *Mitf^mi-vga9/mi-vga9^* mice. *N* = 9 per genotype. The values on the graphs are mean ± SEM DAPI nuclear staining is shown in blue. Scale bars: 50 μm; *p* values were calculated using two-tailed unpaired Student’s *t* test; *p < 0.05.

Histologic analysis showed no detectable defects in the cellular architecture of OBs from P75 to P95 *Mitf* mutant mice (Fig. 2). Expression of TH is reduced upon loss of activity in the OB (Liu et al., 1999) but was not affected in the OBs of *Mitf* mutant mice (Fig. 2*B*,*C*). This also suggests normal OB function. Importantly, *Tbr2*, a marker for M/T cells (Mizuguchi et al., 2012; Imamura and Greer, 2013), is expressed in *Mitf* mutant OBs (Fig. 1*A*) showing normal OB ontogeny. Interestingly, cell counts showed an increase of glomerular Tbr2+ neurons (*t*_(20)_ = 2.914, *p* = 0.0171, Sidak multiple comparison) in the glomeruli of *Mitf* mutant mice (Fig. 1*D*,*E*). An increase in M/T neurons has been shown to occur with increased activity (Johnson et al., 2013; Liu et al., 2016). Additionally, analysis of apoptosis showed a twofold increase in cell death in periglomerular cells of *Mitf* mutant mice (Fig. 3; *t*_(16)_ = 2.724, *p* = 0.015, two-tailed *t* test), suggesting increased glomerular neuronal turnover in the mutant mice.

### *Mitf* mutant mice have reduced I_A_ and a concomitant increase in MC activity

**Figure 4.**
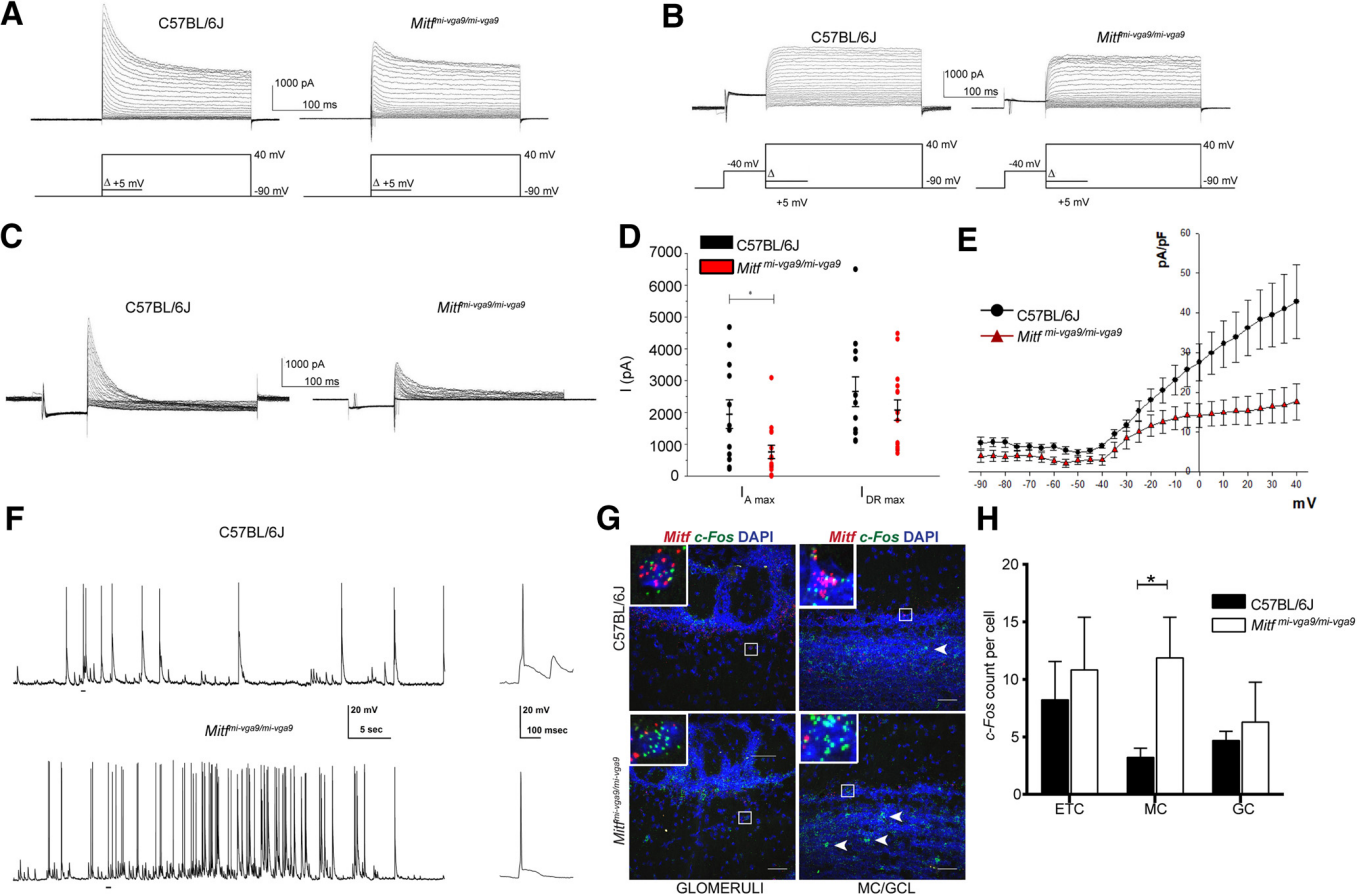
*Mitf* mutant mice have a decrease in I_A_ and a concomitant increase in MC activity. ***A***, Representative images of voltage clamp recordings. ***B***, Representative images of voltage clamp recordings, where the I_A_ was inactivated by a –40-mV prepulse. ***C***, The isolated I_A_ recorded from wild-type and *Mitf^mi-vga9/mi-vga9^* M/T neurons. ***D***, I_A max_ and I_DR max_ in wild type and *Mitf^mi-vga9/mi-vga9^*. *N* = 12 per genotype. ***E***, The relation between current density (pA/pF) of isolated I_A_ currents and membrane voltage (mV) of wild-type and *Mitf^mi-vga9/mi-vga9^* M/T neurons. *N* = 12 per genotype. ***F***, Representative recordings of spontaneous action potentials observed in wild-type (*N* = 5) and *Mitf^mi-vga9/mi-vga9^* M/T neurons (*N* = 3) by current clamp with examples from both recorded traces enlarged on the right. ***G***, RNA *in situ* hybridization of *c-Fos* (green) and *Mitf* (red) in wild-type and *Mitf^mi-vga9/mi-vga9^* OBs. ***H***, *c-Fos* dots per cell in the ETC, MC, and GC. *N* = 4 per genotype. The values on the graphs are mean ± SEM DAPI nuclear staining is shown in blue. Scale bars: 50 μm; *p* values were calculated using two-tailed unpaired Student’s *t* test (***D***), nonlinear regression and one-way ANOVA (***F***) and two-way ANOVA (***H***) and *p < 0.05.

**Figure 5.**
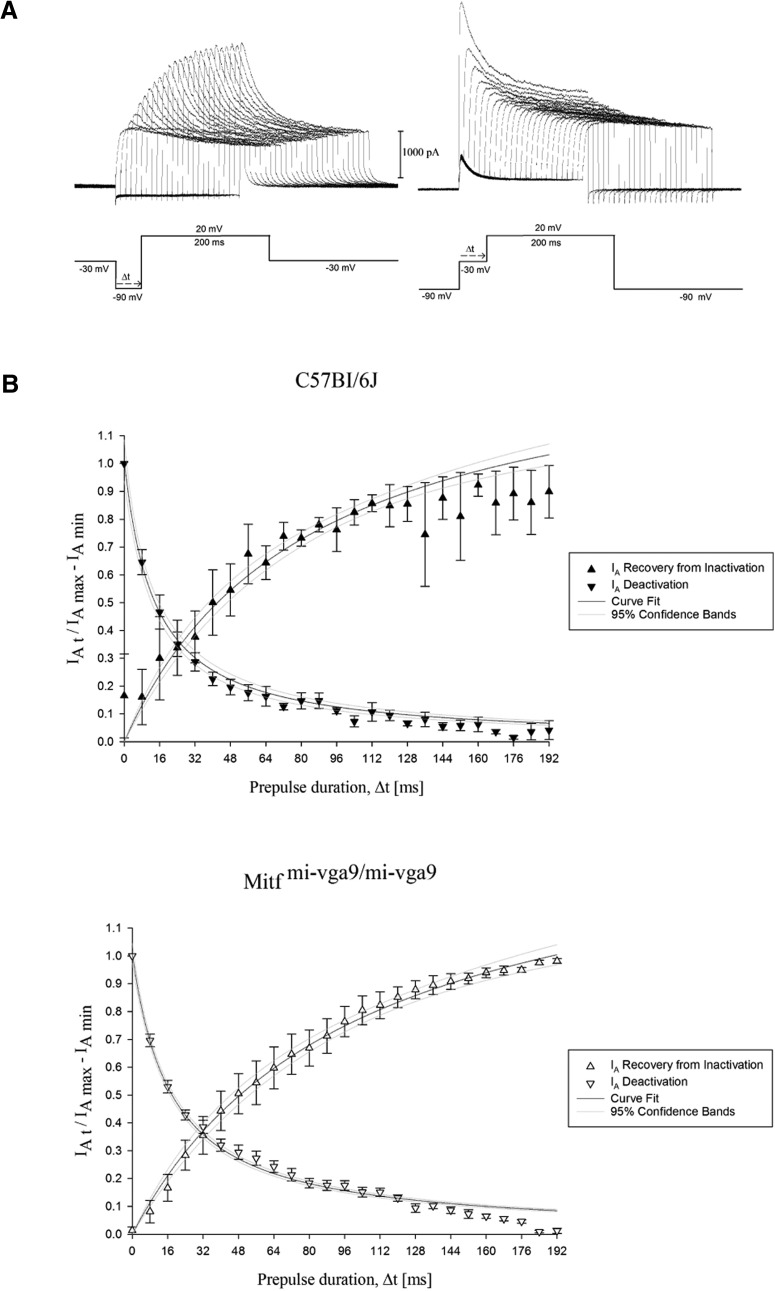
Gating kinetics of the A-type current (I_A_). ***A***, Representative recordings obtained to assess the gating kinetics of the I_A_ current mediating channels. The projection neurons were first voltage clamped at –30 mV and subsequently depolarized to 20 mV using a prehyperpolarizing pulse in between to recover the I_A_ current from inactivation. This was done by increasing the time, Δt, spent in the hyperpolarized state, as indicated by the voltage trace on the left. To gauge its activation kinetics, the neurons were held at –90 mV, as shown in the voltage trace on the right, and subsequently depolarized to 20 mV, with a predepolarization pulse to –30 mV applied in between. The A-type current gets inactivated by increasing the duration of the prepulse, Δt. ***B***, Normalized mean currents (±SEM) plotted separately during recovery of the I_A_ current from inactivation and during deactivation, in cells from wild-type and *Mitf^mi-vga9/mi-vga9^* mice. The functions fitted to the data yield half-activation and half-recovery times of 15.2 ± 4.7 and 42.6 ± 7.2 ms for the wild-type (*N* = 5, *p* = 0.53) and 18.2 ± 1.5 and 51.3 ± 9.3 ms for the Mitf^mi-vga9/mi-vga9^ (*N* = 6, *p* = 0.49) M/T.

To examine whether the loss of *Mitf* resulted in altered M/T neuronal function, we performed whole-cell patch clamp analysis on cultured primary M/T neurons from wild-type and *Mitf* mutant mice, both under voltage clamp and current clamp conditions, bypassing possible secondary effects due to cortical feedback mechanisms or other compensatory mechanisms within the OB. To establish the identity of the cells in culture, several biophysical properties were determined (see methods). The likelihood of an action potential is decided by the balance between sodium influx and potassium efflux. The initial potassium efflux activated near the spiking threshold is termed the A-type potassium current (I_A_) and is a major determinant of the likelihood of an action potential. Patch clamp recordings under voltage clamp showed a reduction in the I_A_ K^+^-current in M/T neurons from *Mitf* mutant mice. (Fig. 4*A–D*; *t*_(50)_ = 2.313, *p* = 0.0491, Sidak multiple comparison). No significant reduction was observed in the I_DR_ current (Fig. 4*D*). There was no significant correlation between the membrane resistance of the M/T neurons and the maximum recorded I_A_ K^+^-current or the I_DR_ current from either wild-type or *Mitf* mutant mice. The I/V relationship denotes the relationship between the I_A_ K^+^-current density (pA/pF) across the membrane and the membrane potential, and thus takes size of the cells into account based on their measured membrane capacitance. The I/V curves show that the mutant neurons have an altered voltage dependence of activation of the I_A_ currents evoked (Fig. 4*E*), with multiple comparisons between the means revealing that the current density is significantly reduced at depolarized membrane potentials, at −5 mV and higher (*p* > 0.05). However, the channel kinetics, i.e., activation and inactivation of the channels mediating the isolated I_A_, remained the same in cells from wild-type and mutant mice (Fig. 5). Reduction in the I_A_ leads to increased likelihood of action potentials and hyperexcitability (Yuan et al., 2005; Kim et al., 2007; Fransén and Tigerholm, 2010). Accordingly, increased spiking was observed in the mutant neurons (Fig. 4*F*), under current clamp configuration of the whole-cell patch clamp. The mean firing frequency of spikes in wild-type M/T neurons was 0.79 ± 0.17 Hz, while it was 1.75 ± 0.15 Hz in mutant M/T neurons (*t*_(11)_ = 1.61, *p* = 0.007, two-tailed *t test*). The mean amplitude of action potentials was similar, or 62 ± 4 mV in wild-type M/T neurons and 54 ± 2.6 mV in mutant M/T neurons (*t*_(11)_ = 1.06, *p* = 0.31, two-tailed *t test*). In addition to spikes, spontaneous excitatory postsynaptic potentials (EPSP) could be observed in the current clamp recordings. Additionally, expression of *c-Fos*, an activity-dependent gene frequently used to monitor neuronal activity, was increased in the *Mitf* mutant MCs (Fig. 4*G*,*H*; *t*_(9)_ = 3.093, *p* = 0.0386, Sidak multiple comparison), indicating increased neuronal activity *in vivo*. A second, unidentified cell population in the GCL also showed clear increase in expression of *c-Fos* in the mutant (Fig. 4*G*, arrowheads).

### Expression of *Kcnd3* and *Mitf* is activity dependent in the OB

**Figure 6.**
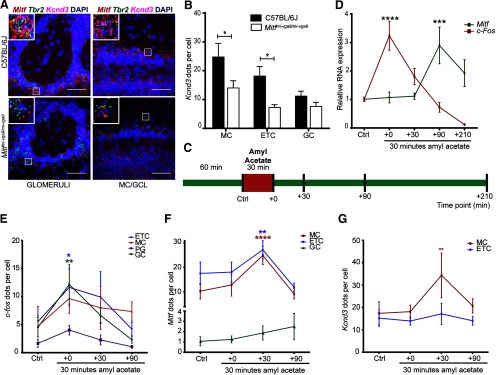
*Kcnd3* and *Mitf* expression are activity dependent in the OB. ***A***, RNA *in situ* hybridization of *Tbr2* (green), *Mitf* (red), and *Kcnd3* (magenta) performed on wild-type and *Mitf^mi-vga9/mi-vga9^*OBs. ***B***, *Kcnd3* count per cell. *N* = 5 per genotype. ***C***, Schematic representation of the amyl acetate (AA) experiment where mice were habituated to an odorless cage for 60 min. Upon exposure to AA for 30 min (red block), they were sacrificed at different time points (black vertical lines). ***D***, *Mitf* and *c-Fos* mRNA expression in wild-type OB following AA, determined by RT-qPCR. *N* = 6 per time point. ***E***, *c-Fos* dots per cell in wild-type OB mice following AA. *N* = 6 per time point. ***F***, *Mitf* dots per cell in wild-type OB following AA. *N* = 6 per time point. ***G***, *Kcnd3* dots per cell in wild-type OB following AA. *N* = 6 per time point. The values on the graphs are mean ± SEM DAPI nuclear staining is shown in blue. Scale bars: 50 μm; *p* values were calculated using two-way ANOVA (***B***, ***D***, ***F***, ***F***, ***I***); *p < 0.05, **p < 0.01, ***p < 0.001, ****p < 0.0001. PG = periglomerular.

**Figure 7.**
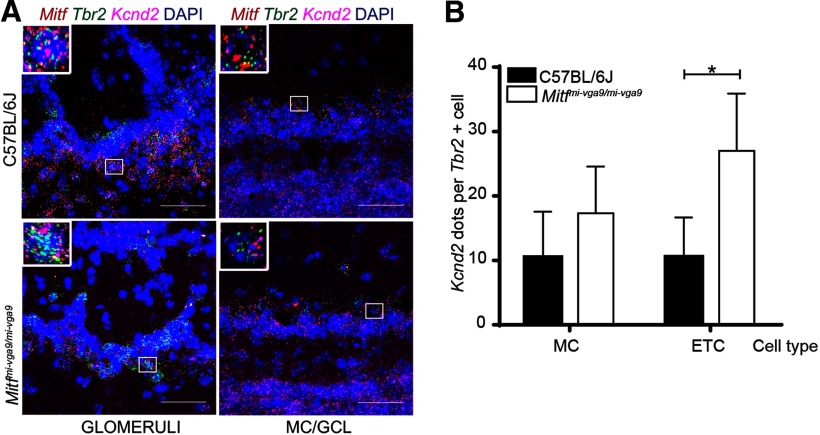
*Kcnd2* expression is increased in the *Mitf* mutant OB. ***A***, RNA *in situ* hybridization of *Tbr2* (green), *Mitf* (red), and *Kcnd2* (magenta) performed on wild-type and *Mitf^mi-vga9/mi-vga9^*OBs. ***B***, *Kcnd3* count per cell. *N* = 4 per genotype. The values on the graphs are mean ± SEM DAPI nuclear staining is shown in blue. Scale bars: 50 μm; *p* values were calculated using two-way ANOVA (***B***); *p < 0.05.

Increased neuronal activity due to the loss of I_A_ led us to examine the expression of key potassium channel subunits in the OB. As displayed in Table 1, many potassium channel subunits (Gutman et al., 2005) are known to be expressed in the OB. We focused our analysis on the I_A_-current regulating Kv4.3/*Kcnd3* potassium channel subunit (Kollo et al., 2008; Wang et al., 2015), as according to ChIPseq data, MITF binds to its regulatory region (Table 1; Strub et al., 2011; Calero-Nieto et al., 2014; Laurette et al., 2015). RNA *in situ* analysis showed reduced expression of *Kcnd3* in ETCs (*t*_(12)_ = 3.297, *p* = 0.0190, Sidak multiple comparison) and MCs (*t*_(12)_ = 3.249, *p* = 0.0208, Sidak multiple comparison) of *Mitf* mutant OBs, whereas the levels remained similar in cells of the GCL (Fig. 6*A*,*B*). Thus, the effects of *Mitf* on *Kcnd3* expression are cell autonomous, consistent with the possibility of *Mitf* regulating *Kcnd3* in M/T neurons. Although the ChIPseq data does not show MITF to bind to the regulatory region of the gene encoding the I_A_-regulating potassium channel subunit *Kcnd2* (Table 1), its expression was increased in ETC in OB from the *Mitf* mutant mouse (*t*_(6)_ = 5.051, *p* = 0.0047, Sidak multiple comparison), possibly as a compensatory mechanism for the reduction in *Kcnd3* (Fig. 7). This increase in the expression of *Kcnd2* underlines the tight regulation of neuronal activity. Without this change, the effects on the A-current would likely be larger, especially in the ETC.

**Table 1:** Potassium channel subunits in the OB

Gene	K^+^- channelsubunit	ChIPseq peak^1^				ABA expression in OB^2^	Current^3^
		Strub^4^2011	Laurette^5^2015	Young^6^2014	Calero-Nieto^7^ 2014		
*Kcna1*	Kv1.1	No	No	No	No	Low expression in GCL	I_DR_
*Kcna2*	Kv1.2	Yes	Yes	No	No	Expressed in MCL and ETC	I_DR_
*Kcna3*	Kv1.3	No	Yes	No	No	Expressed in MCL and ETC	I_DR_
*Kcna4*	Kv1.4	No	No	No	No	Expressed in MCL, ETC and GCL	I_A_
*Kcna5*	Kv1.5	No	No	No	No	Low expression in ETC	I_A_ with β-subunitKvB3
*Kcnc1*	Kv3.1	No	No	No	No	Expressed in MCL and ETC	I_DR_
*Kcnc2*	Kv3.2	No	No	No	No	Expressed in PGC, ETC, MCL	I_DR_
*Kcnc3*	Kv3.3	No	No	No	No	Expressed in MCL and ETC	I_A_
*Kcnc4*	Kv3.4	No	No	No	No	Low expression in MCL	I_A_
*Kcnd1*	Kv4.1	No	No	No	No	Expressed throughout the OB	I_A_
*Kcnd2*	Kv4.2	No	No	No	No	Expressed in MCL and GCL	I_A_
*Kcnd3*	Kv4.3	Yes	Yes	No	No	Expressed throughout the OB	I_A_

Available ChIPseq data, Allen Brain Atlas expression of potassium channel subunits in the OB and published data on the type of current these subunits are regulating.

1 “No” indicates that no peak observed was higher than negative control, whereas “yes” indicates that the peak observed was higher than the negative control. Region screened encompassed whole intronic regions and approximately 2 kbp up and downstream of transcription start site.

2 GCL, PGC = periglomerular cells.

3 I_DR_ = delayed rectifying current, I_A_= A-type current Gutman et al. (2005).

4 GEO Accession number GSE64137.

5 GEO Accession number GSM1517751.

6 GEO Accession number GSM1567047.

7 GEO Accession number GSM1167584.

**Figure 8.**
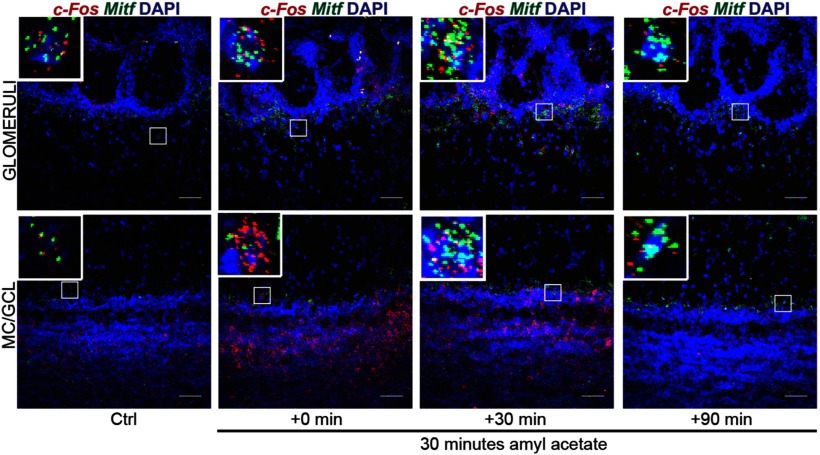
*c-Fos* and *Mitf* expression is increased upon activity induction in the OB. RNA *in situ* hybridization of *c-Fos* (red) and *Mitf* (green) performed on wild-type OBs following AA. DAPI nuclear staining is shown in blue. Scale bars: 50 μm.

**Figure 9.**
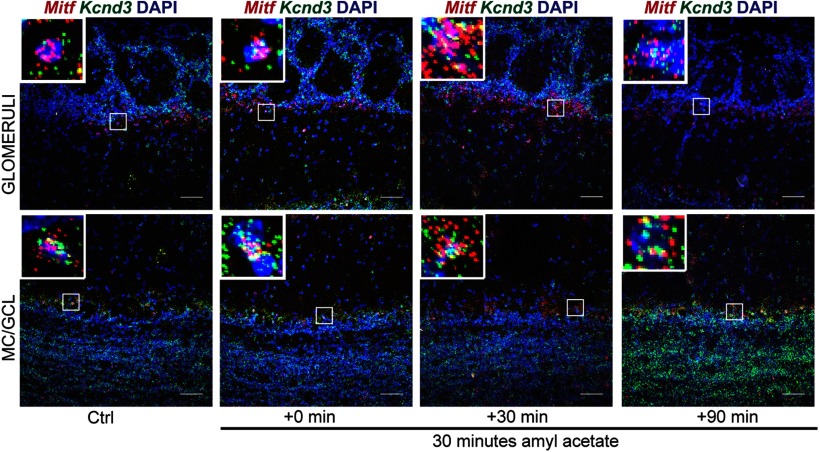
Kcnd3 expression is increased on activity induction in the OB. RNA *in situ* hybridization of *Mitf* (red) and *Kcnd3* (green) performed on wild-type OBs following AA. DAPI nuclear staining is shown in blue. Scale bars: 50 μm.

Neuronal activity leads to generation of cAMP, activation of the MAPK pathway kinases and the cAMP-responsive element binding protein (CREB) protein. These are established regulators of *Mitf* transcription and MITF signaling (Shoag et al., 2013). We therefore explored the effects of OSN activity on the expression of *Mitf* and *Kcnd3* in the OB. Amyl acetate is known to activate a multitude of glomeruli (Bepari et al., 2012). Amyl acetate treatment of the wild-type mice (Fig. 6*C*) resulted in an immediate increase in the global transcription of *c-Fos* (Figs. 7*E*, 8; *q*_(40)_ = 4.875, *p* < 0.0001, Dunnett’s multiple comparison) as expected. Similarly, RNA *in situ* hybridization showed significant cell-specific increase of *c-Fos* in the ETCs (*q*_(57)_ = 2.687, *p* = 0.0241, Dunnett’s multiple comparison) and GCs (*q*_(57)_ = 3.359, *p* = 0.0036, Dunnett’s multiple comparison) with treatment. An increase was also observed in *c-Fos* expression in MCs, but this was not significant (*q*_(57)_ = 2.365, *p* = 0.0561). Note, however that not all glomeruli are affected by amyl acetate (Bepari et al., 2012). The increase in *c-Fos* expression was followed by an increase in *Mitf* expression 30 min after exposure to amyl acetate both in the ETCs (Figs. 6*F*, 8; *q*_(45)_ = 3.325, *p* = 0.005, Dunnett’s multiple comparison) and the MCs (*q*_(45)_ = 5.031, *p* = 0.0001, Dunnett’s multiple comparison). Expression of *Kcnd3* also increased 30 min after exposure, but only in the MCs (Figs. 6*G*, 9; *q*_(30)_ = 3.271, *p* = 0.0075, Dunnet’s multiple comparison) and not in the ETCs. This showed that expression of both *Mitf* and *Kcnd3* is activity dependent in the MC projection neurons of the OB. The large increase observed for *Mitf* expression after 90 min (Fig. 7*D*) suggests that *Mitf* is also activity dependent in the GCL, but the GCs greatly outnumber M/T neurons.

### Activity-dependent increase in *Kcnd3* expression requires MITF enhancer activity

**Figure 10.**
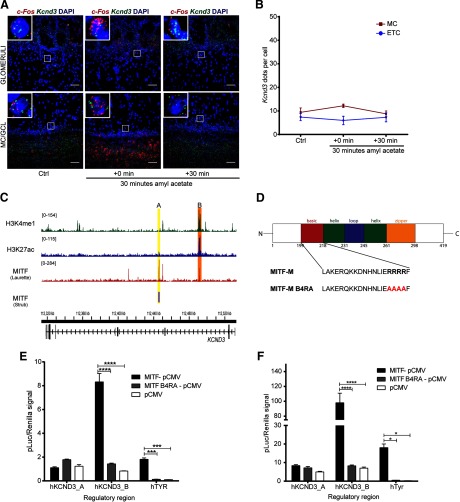
Activity-dependent increase in *Kcnd3* expression requires MITF enhancer activity. ***A***, RNA *in situ* hybridization of *c-Fos* (red) and *Kcnd3* (green) performed in *Mitf^mi-vga9/mi-vga9^* OB following AA. ***B***, *Kcnd3* dots per cell in *Mitf^mi-vga9/mi-vga9^* OB following AA. *N* = 3–5 per time point. ***C***, ChIPseq peaks of MITF, H3K27ac and H3K4me1 on *KCND3* gene in 501mel cells. The MITF peaks are labeled A (orange) and B (yellow). Yellow shows overlapping peak of MITF binding in Strub and Laurette datasets (*B*), whereas orange indicates MITF peaks that overlap with H3K27ac and H3K4me1 peaks (*A*). ***D***, Sequence of the basic region of wild-type (MITF-*M*) and transcriptionally inactive MITF with four argenines mutated to alanines (MITF-M B4RA). ***E***, Transcription activation assays performed in HEK293T cells co-transfected with constructs containing the hTYR, hKCND3_pA, and hKCND3_pB regulatory regions fused to luciferase, together with empty vector or wild-type or transcriptionally inactive (B4RA) MITF constructs. *N* = 3. ***F***, Transcription activation assays performed in N2A cells co-transfected with constructs containing the hTYR, hKCND3_pA, and hKCND3_pB regulatory regions fused to luciferase, together with empty vector or wild-type or transcriptionally inactive (B4RA) MITF constructs. *N* = 3. The values on the graphs are mean ± SEM DAPI nuclear staining is shown in blue. Scale bars: 50 μm; *p* values were calculated using two-way ANOVA (***B***, ***E***, ***F***); *p < 0.05, ***p < 0.001, ****p < 0.0001.

Induction of large-scale OB activity with amyl acetate was also performed on *Mitf* mutant mice. The increase observed in *Kcnd3* expression upon inducing neuronal activity in wild-type OBs was not observed in *Mitf* mutant OBs (Fig. 10*AB*). Hence, *Mitf* and *Kcnd3* are both regulated in an activity-dependent manner in the MC and the expression of *Kcnd3* depends on *Mitf* in both MC and ETC. Analysis of two MITF ChIPseq datasets (Strub et al., 2011; Laurette et al., 2015) showed that MITF binds to intronic regions of *KCND3* in 501mel melanoma cells. The MITF ChIPseq datasets show the same overlapping binding peak B (Fig. 10*C*, orange). A second MITF binding peak, peak A, was observed only in the Laurette dataset and overlaps with H3K27ac and H3K4me1 ChIPseq peaks in 501mel melanoma cells (Fontanals-Cirera et al., 2017) indicative of an active enhancer (Fig. 10*C*, yellow). Transcription activation analysis showed that MITF activates expression from a fragment containing peak B in both HEK293T cells (Fig. 10*E*; MITF-pCMV vs pCMV: *q*_(12)_ = 22.58, *p* = 0.0001; MITF-pCMV vs MITFB4RA-pCMV: *q*_(12)_ = 20.78, *p* = 0.0001, Dunnett’s multiple comparison) and N2A cells (Fig. 10*F*; MITF-pCMV vs pCMV: *q*_(12)_ = 15.39, *p* = 0.0001; MITF-pCMV vs MITFB4RA-pCMV: *q*_(12)_ = 15.18, *p* = 0.0001, Dunnett’s multiple comparison), whereas transcriptionally inactive MITF does not (Fig. 10*E*,*F*). *Tyr* was used as a positive control and was significantly activated in both cell types. No activation was observed in either cell type from a fragment containing peak A. As the region around peak B has active enhancer marks, we conclude that MITF signaling is induced by activity and regulates an enhancer region in an intron of *Kcnd3* leading to an activity-dependent increase in *Kcnd3* expression in projection neurons of the OB. In accordance with this, stimulus dependent enhancer activity has previously been described in neurons (Walke et al., 1996; Kim et al., 2010; Guzman-Karlsson et al., 2014; Malik et al., 2014; Vierbuchen et al., 2017).

### Loss of *Mitf* affects long-term adaptation

**Figure 11.**
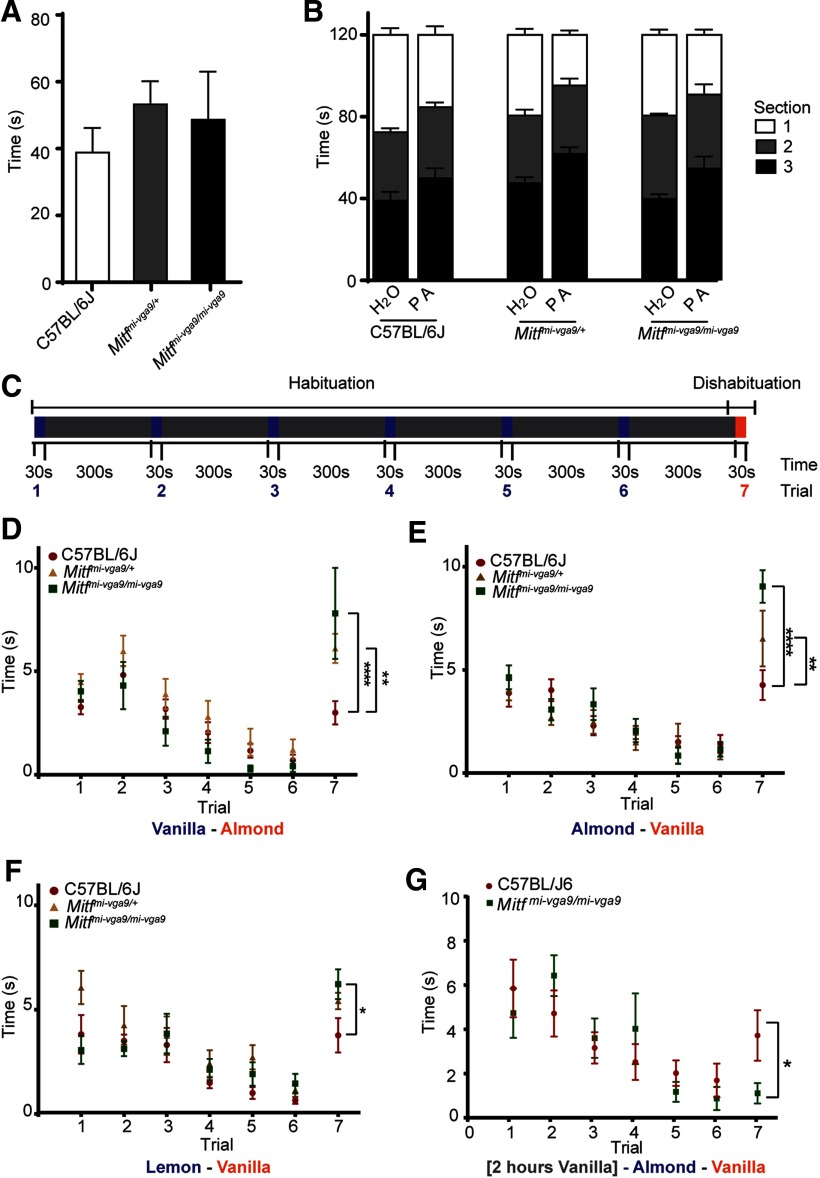
*Mitf* regulates olfactory discriminatory ability and odor fatigue recovery. ***A***, Quantification of the results of the hidden cereal odor detection-assay. *N* = 8 per genotype. ***B***, Avoidance assay where a cage was sectioned in three equal areas and mice were exposed to water or propionic acid (PA) in section 1. Quantification shows time spent in each section (Table 2). *N* = 6–8 per genotype. ***C***, Schematic overview of the dishabituation-habituation assay, where mice are exposed to odor A (blue) for 30 s six times with 5-min intervals, after which they are exposed to odor B (red) for 30 s. ***D***, Dishabituation-habituation, mice were exposed to vanilla as odor A and almond as odor B. *N* = 8 per genotype. ***E***, Dishabituation-habituation, where mice were exposed to almond as odor A and vanilla as odor B. *N* = 6–8 per genotype. ***F***, Dishabituation-habituation, mice were exposed to almond as odor A and lime as odor B. *N* = 8 per genotype. ***G***, Dishabituation-habituation assay where the mice were exposed to vanilla for 2 h, followed by almond as odor A and vanilla as odor B. *N* = 15 per genotype. The values on the graphs are mean ± SEM; *p* values were calculated using one-way ANOVA (***A***) or two-way ANOVA (***B***, ***D–G***); *p < 0.05, **p < 0.01, ****p < 0.0001.

Hyperactive MCs are likely to affect olfaction. Changes in the olfactory neuronal circuits frequently lead to reduced ability to detect odorants or to differentiate between them. Both are critical functions of the olfactory system. Olfactory testing, however, showed no difference in the ability of *Mitf* mutant and wild-type mice to detect or avoid odors (Fig. 11*A*,*B*; Table 1). Next, we tested the ability of *Mitf* mutant mice to differentiate between odorants. Repeated introduction of the same odorant leads to reduced interest (habituation) and when followed by a novel odorant, renewed interest (dishabituation; Fig. 11*C*), an established measurement of reduced olfactory discriminatory ability (Yang and Crawley, 2009). In this assay, the *Mitf* mutant mice showed increased dishabituation when exposed to the chemically similar odorant pair vanilla-almond (Fig. 11*D*; *t*_(84)_ = 5.223, *p* < 0.0001, Dunnett’s multiple comparison). This was also observed when the odorants were introduced in the reverse order (Fig. 11*E*; *t*_(126)_ = 5.838, *p* = 0.0001, Dunnett’s multiple comparison) and when the less chemically similar odorant pair lemon-vanilla was used (Fig. 11*F*; *t*_(147)_ = 2.708, *p* = 0.0144, Dunnett’s multiple comparison). This can be interpreted as increased olfactory discriminatory ability or increased olfactory sensitivity. It is also possible that the mutant mice are more interested in the novel, second odorant. Importantly, *Mitf^mi-vga9/+^* heterozygotes, which are phenotypically similar to wild-type mice, show an intermediate phenotype (Fig. 11*D–F*). This demonstrates that the changes in olfaction in the *Mitf* homozygotes are not due to larger cortical area devoted to olfactory processing caused by their blindness and deafness. Odor exposure reduces sensitivity of M/T neurons (Kato et al., 2012) and the appropriate long-term adaptation to sensory input is fundamental to olfaction. Our results suggest that the mutant mice might not adapt well on a longer timescale, as *Mitf*-dependent transcription would be required. We therefore tested the effects of a long-term exposure of an odorant on olfactory ability, also termed odor fatigue. Under normal circumstances this leads to a reduction in ability to detect the odorant (habituation), followed by recovery once the odorant is removed. However, in the mutant mice, there was a stark reduction in the ability to detect the odorant as six out of eight mutant mice tested did not detect the initial odor when reintroduced (Fig. 11*G*; *t*_(84)_ = 3, *p* = 0.0246, Sidak’s multiple comparison). As this odorant is novel when reintroduced, this makes it unlikely that the *Mitf* mutant mice are only more interested in new odors. Both assays thus indicate clear differences in olfactory mechanisms between wild-type and mutant mice.

**Table 2:** Two-tailed paired Student’s *t* test performed on avoidance behavior

	C57Bl/6J	*Mitf^mi-vga9/+^*	*Mitf^mi-vga9/mi-vga9^*
Section 1	0.0016*	0.0009*	0.0004*
Section 2	0.6943	0.9325	0.3859
Section 3	0.0408*	0.0138*	0.0267*

Two-tailed paired Student’s *t* test performed on the time mice spent in each section, comparing water and propionic acid conditions; *p < 0.05; *N* = 6–8.

**Figure 12.**
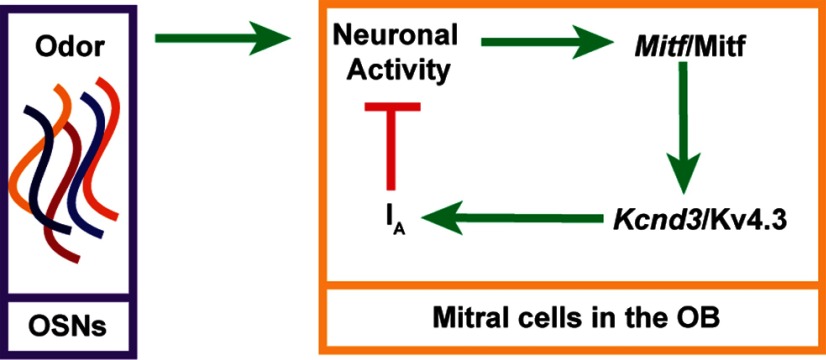
Model of MITF as a regulator of homeostatic intrinsic plasticity. Upon neuronal activity in the OB, MITF is required for an increase in *Kcnd3* expression. More Kv4.3 protein results in an increase in the I_A_ potassium current and reduced likelihood of action potentials, subsequently leading to decreased activity of M/T cells.

We propose a hypothesis for a functional role of *Mitf* in projection neurons of the OB, where it regulates the intrinsic excitability of the neurons following activity induction, by reducing the neuronal firing rate of neurons through its regulation of *Kcnd3* expression. In this model, the activity of OSNs leads to an increase in *Mitf* expression, and an *Mitf*-dependent increase in the transcription of *Kcnd3* in the output neurons of the OB. It is possible that the presence of MITF, rather than its level or postranslational state, guides the expression of *Kcnd3* following activity. An increased I_A_-current decreases the likelihood of the generation of action potential and reduces activity leading to decreased sensitivity of the glomeruli in question (Fig. 12). Conversely, lack of activity in a glomerulus could lead to the opposite mechanism and increased sensitivity (Tyler et al., 2007).

## Discussion

The transcription factor *Mitf* is expressed distinctly in the excitatory neurons (M/T) of the mouse OB (Ohba et al., 2015). M/T neurons are generated in the *Mitf* mutant mouse and express the appropriate neuronal markers suggesting that loss of *Mitf* does not affect their development. However, electrophysiological analysis of M/T cells *in vitro* showed that these cells are hyperactive and show loss of the potassium I_A_-current. This is further supported by reduced gene expression of *Kcnd3*, a potassium channel subunit required for the I_A_-current. Expression of the activity-dependent *c-Fos* gene showed that there is an increase in neuronal activity in *Mitf* mutant mice *in vivo*. However, this was not observed in all M/T cells. ETCs, a subclass of tufted cells, which drive olfactory output (Hayar et al., 2004a) and link OSN with M/T neurons (Gire et al., 2012), do not show increased expression of *c-Fos* under baseline conditions in the mutant. On the other hand, MCs, the key projection neurons of the OB, showed increased expression of *c-Fos* in *Mitf* mutant OBs. Interestingly, by inducing olfactory activity in wild-type mice using a strong odorant known to activate many glomeruli, we observed an increase in both *Mitf* and *Kcnd3* expression in the MCs. The expression of both *c-Fos* and *Mitf*, but not *Kcnd3*, was increased upon activity in the ETC. This demonstrates that the MCs and ETCs, which have different functions in the olfactory neuronal circuit, also have a different intrinsic response to activity. One possible interpretation is that the transcription of *Kcnd3* is not regulated by *Mitf* in the ETCs during activity as the adaptive response is more efficient or faster when it occurs only in the key output neurons, rather than also in neurons which drive their activity. It is also noteworthy that ETC show increased expression of *Kcnd2* in the mutant (Fig. 7), which would dampen the effect of reduced *Kcnd3* and thus explain reduced effect on ETC’s activity.

The increased activity of projection neurons has wide-ranging consequences for OB function. The *Mitf* mutant mice show increased olfactory dishabituation, which might mean increased ability to discriminate between odors or increased olfactory sensitivity. This is a rarely reported characteristic of mutant mice (Fadool et al., 2004), and the expectation is that olfaction works at maximum discriminatory power, since this is most beneficial for the organism. As we detect no changes in mobility, motivation nor interest in novelty in other assays (Fig. 11*A*,*B*,*G*), we conclude that this phenotype is inherent to the OB, also due to the fact that the heterozygote shows an intermediate phenotype. One explanation, for this difference in dishabituation is the increased number of glomerular excitatory neurons of the OB. The nature of the olfactory phenotype in the *Mitf* mutant mouse is also likely due to increased levels of lateral inhibition due to increased activity (Nunez-Parra et al., 2013; Nunes and Kuner, 2015) and/or more glomeruli being activated by each odorant due to lower threshold of firing, leading to reduced cross-habituation (Mombaerts et al., 1996). Further studies will determine this. Importantly, while the *Mitf* mutant mouse has increased dishabituation, long-term odor exposure leads to a reduction in the ability to detect that odorant, i.e., increased habituation. This shows that *Mitf* is required for normal long-term adaptation in the OB and that the increase in olfactory sensitivity observed in the mutant mice comes at a cost. Likely functional explanations are either that projection neurons are overwhelmed with activity or that high activity of projection neurons in the mutant leads to stronger adaptation in the olfactory cortex. Both cases show how important *Mitf* is for olfactory adaptation.

Neuronal activity sculpts the nervous system and is required for adaptive changes in neuronal function. To do so, neuronal activity must functionally change the neuron, by impacting its firing frequency or the strength of individual synapses. However, neurons also need to tightly regulate their own activity: too much neuronal activity is costly and can lead to excitotoxicity or uncontrolled firing, whereas the loss of neuronal activity leads to loss of information and neurodegeneration. To avoid this, mechanisms are in place to maintain neuronal activity within a set window, and the ability of a neuron to be plastic has to be within the confines of such neuronal homeostasis (Turrigiano, 2008; Pozo and Goda, 2010; Titley et al., 2017). Our results show how sensory activity can affect firing rate of the OB projection neurons in an *Mitf* dependent manner through effects on A-type currents via *Kcnd3* expression. This mechanism can maintain neuronal homeostasis or determine the sensitivity or neuronal gain-control of the projection neurons on a longer time scale, i.e., it is based on transcriptional changes. Importantly, electrophysiological analysis in primary neurons shows that changes in potassium currents are cell autonomous and not due to changes in OB circuits. Detailed analysis of the electrophysiology of M/T neurons in OB slices and *in vivo* is needed to further dissect the consequences for olfaction.

Sensory systems focus on detecting novelty or change. In olfaction, this translates into the ability to quickly distinguish rare but important odors from background odors (Tyler et al., 2007; Kato et al., 2012). In the OB, *Mitf* is pivotal to the adaptation of individual glomeruli through regulating intrinsic homeostatic plasticity in an activity-dependent manner. Most glomeruli are activated rarely by odorants. However, they can play important roles in detecting rare threats or food sources, both of high survival value. Keeping these glomeruli sensitive is therefore of high adaptive value and our model provides a mechanism through which this can occur. Thus, regulation of intrinsic plasticity, as described here, provides a mechanism through which olfaction can be insensitive to background odors but sensitive to rare odors.

## Synthesis

Reviewing Editor: Alfonso Araque, University of Minnesota Decisions are customarily a result of the Reviewing Editor and the peer reviewers coming together and discussing their recommendations until a consensus is reached. When revisions are invited, a fact-based synthesis statement explaining their decision and outlining what is needed to prepare a revision will be listed below. The following reviewer(s) agreed to reveal their identity: Mihaly Kollo, Mickey London.

The reviewers have found merit and interest on the findings reported. I concur with the reviewers’ comments expressing the interest of the study. However, they also expressed some concerns about some specific issues that need to be addressed and clarified (see below the specific comments). Importantly, both reviewers found some conclusions to be overreaching, and suggest that claims need to be adjusted to the data according to their comments and re-frame the conclusions to make the manuscript less speculative. Otherwise, you will need to provide additional experimental evidence and analysis as indicated in the reviewers’ comments. I also consider pertinent these concerns.

The time required to address the reviewers´ concerns is estimated to be less than 2 months’ work.

I am looking forward to receiving the revised version of the manuscript.

Best regards,

Alfonso

Specific comments of the reviewers:

Reviewer #1

This paper addresses an important question regarding the activity-dependent regulation of active conductances in olfactory bulb neurons. It confirms previous results that Microphthalmia-associated transcription factor (Mitf) is expressed in olfactory mitral and tufted cells and demonstrates that Mitf (mi-vga9) mutant mice have reduced expression of Mitf in these neurons, while the gross morphology of the circuit is preserved. They show a two-fold increase in the apoptosis of periglomerular cells in Mitf(mi-vga9), a reduction of A-type current and increased c-fos expression and spontaneous spike rate in M/T cells.

The study further shows that Mitf is activity-dependent: Mitf expression increases after odor exposure. The paper provides evidence that Mitf regulates an enhancer region of Kcnd3.

Finally, in behavioral experiments, Mitf mutant mice showed increased dishabituation for odors. The paper proposes a mechanism for the observed changes, where neuronal activity increases MITF expression that in turn leads to increased Kcnd3 expression and the reduced excitability of the mitral and tufted cells.

The experiments addressing the expression of Mitf and related changes in A-type potassium channels are well performed and described. While the influence of Mitf on Kcnd3 expression is well supported by evidence, a change in intrinsic properties as a link between Mitf expression and behavioral changes, while plausible, is less convincingly demonstrated. It also has to be noted, that the electrophysiological experiments operate with small sample sizes and the p-values are just below threshold.

Major points:

1) While the study demonstrates a change in isolated A-type currents in M/T cells convincingly, apart from the spontaneous spike rate, there is very little evidence presented for altered active properties in those cells. The probability of spiking upon current injection at different amplitudes in current-clamp configuration, for example, could have been tested, and it is unclear why this, or other more direct measures of excitability were not shown.

2) In the behavioral dishabituation assay, significant changes are shown in Mitf(mi-vga9/+) heterozygotes. However, there is no mention in the text of any change in the potassium channel expression in heterozygotes. This would be an important piece of information to confirm that the observed behavioral changes are related to Kcnd expression in the olfactory bulb mitral cells.

3) The author’s hypothesis regarding ET cells is unclear. While their model is presented for projection neurons in general, ET cells are certainly not following the proposed mechanism in Figure 6. While there is an activity-dependent increase of Mitf in those cells, as well a significant effect of Mitf on Kcnd3 expression, there is no activity-dependent regulation of Kcnd3. This is especially difficult to interpret, as ETCs have a bigger drop in Kcnd3 in Mitf mutants than MTCs.

The paper should present a hypothesis of how the observed effects in ETCs are compatible with the proposed mechanism.

4) The compensatory increase in Kcnd2 presented in Figure 10 is on par, if not stronger than the decrease of Kcnd3 expression in Mitf(mi-vga9/mi-vga9) animals. It is unclear, and the authors should present a reasonable explanation, how this doesn’t prevent the loss of A-type currents in M/T cells.

5) The spike amplitudes displayed on the current clamp traces of Figure 2 are very small: The Results section mentions amplitudes of 54 and 62 mV, while the example traces on Figure 2f displays less than 30 mV spikes measures from baseline in wild type, and barely over 30 mV in mutants as a representative example, which raises the question whether spontaneous spike rates could have been influenced by variability in patch-clamp recording quality.

At the displayed time scale, it is difficult to assess spike shape, but the lack of afterhyperpolarization, and the fast time course of EPSPs, taken together with the small spike amplitude, are much more characteristic of granule cells than mitral cells. The authors should display a blown-up version of the recorded traces to address these concerns and allow a better assessment of the spike waveform.

Minor points:

1) Page 18, last paragraph: Evidence for Kv4 protein expression on the M/T cell plasma membrane at the protein level is available in published literature and could be cited here (e.g. Wang et al. 2015 J Comp Neur and Kollo et al. 2008 Eur J Nsci).

2) Page 18, last paragraph: How were the cell boundaries defined when counting fluorescent dots?

3) Figure 5c: The colors of the plot (blue and red) do not match the colors mentioned in the legend (red and green).

4) Multiple locations in the text: when discussing spontaneously occurring EPSPs, the expression “miniature EPSP” is used. This is generally used for recordings done in TTX, where release is AP independent. “Spontaneous EPSP” would be a more appropriate expression.

Reviewer #2

This manuscript studies the interaction between the neuronal activity of specific neurons in the olfactory bulb and the expression of MITF. The thesis of the manuscript is that the activity of projection neurons in the OB drives the expression of MITF, which in turn regulates the potassium current I­A through the regulation of the subunits Kcnd3/Kv3.4. This, in turn, affects the sensitivity of the neurons to their synaptic inputs and thus acts as a homeostatic mechanism to regulate the activity of projection neurons of the OB.

I find the paper written well and interesting. I am not an expert in some of the methods used by the authors (mainly mFISH and molecular biology), but I find them adequate to support their point by point conclusions.

However, while each of the points is well made, there are some significant gaps in the story as a whole. For example:

There is no demonstration of changes in the activity of OB projection neurons in vivo in the mutant mice. I appreciate that the authors wanted a clear demonstration of the effect of the mutation on activity on the specific cells, which is right and nice, but if there are some other mechanisms that change the activity in vivo, then the argument that MITF is the regulator is weaker. So when in figure 5, the author assumes that there are changes in activity that cause the change in behavior, the argument would be much stronger if they would show those changes in the activity.

Similarly, the authors only study the effect of the mutant (i.e., reduction in MITF), but they do not show the effect of elevation in MITF. However, they feel comfortable speculating that an increase in MITF will cause a reduction in activity (pp 22) even though this was not demonstrated in the data at all. For now, as far as I can see, they can only claim one direction of the cycle but not the complete one.

Similarly, the mutant is not targeted only to the OB. So, while there are clear changes in the OB due to the mutation, the authors state that there are other effects (e.g., microphthalmia), so the change in behaviour may result from other effects of MITF in other neuronal systems just as well.

In conclusion, I find the study compelling and interesting, but the different levels or experiments are not tight together very strongly. I suggest that the authors will take one of two paths. One, make changes to the text to better reflect the relationship between what they find and what they claim or Two, add more intermediate level experiments such as overexpressing MITF and/or recording activity in the OB in vivo in the mutant and strengthen their support of the claims.

Please see more specific comments below.

Specific Comments:

Figure 2A-D:

The difference between WT and mitf mutant look convincing, but the statistics are really borderline (0.049). Why is that?

In both B and C the recordings from the mutant, but not WT, show at least one example of an escape current (un-clamped spike?). This suggests that the VC is compromised by the cell geometry (the current source is away from the recording location). The authors should provide an image or reconstruction of the recorded cell to show that there is no outstanding morphological difference between the WT and mutant (I don’t think there is a need for detailed morphological comparison but just a bold eye comparison).

The analysis of mEPSPs on pp18 is confusing. First, it looks like that they are not minis and but rather spontaneous (i.e., triggered by presynaptic spikes). Secondly, if the firing frequency of all cells increases, one would expect an increase in the frequency of these EPSPs, which you don’t find. Thirdly, one possible reason for the insignificance you see is because they might trigger APs, and then you don’t count them. It looks like this analysis should either be dropped or done on EPSCs (in VC) and not in CC.

I have little experience with the behavioural results presented in figure 5D-G. They clearly are significant, but the effects, for an inexperienced eye, seems rather weak.

Minor:

1. Supp figures are referenced by regular numbers, which creates confusion. For example, Pp16 - figure 7 is referenced straight after figure 1, and on pp17 figure 2 comes and then figure 9. Can’t you use figure 1, figure s1, figure 2, figure s2 ...

## Author Response

Specific comments of the reviewers with replies:

Reviewer #1

This paper addresses an important question regarding the activity-dependent regulation of active conductances in olfactory bulb neurons. It confirms previous results that Microphthalmia-associated transcription factor (Mitf) is expressed in olfactory mitral and tufted cells and demonstrates that Mitf (mi-vga9) mutant mice have reduced expression of Mitf in these neurons, while the gross morphology of the circuit is preserved. They show a two-fold increase in the apoptosis of periglomerular cells in Mitf(mi-vga9), a reduction of A-type current and increased c-fos expression and spontaneous spike rate in M/T cells.

The study further shows that Mitf is activity-dependent: Mitf expression increases after odor exposure. The paper provides evidence that Mitf regulates an enhancer region of Kcnd3. Finally, in behavioral experiments, Mitf mutant mice showed increased dishabituation for odors. The paper proposes a mechanism for the observed changes, where neuronal activity increases MITF expression that in turn leads to increased Kcnd3 expression and the reduced excitability of the mitral and tufted cells.

The experiments addressing the expression of Mitf and related changes in A-type potassium channels are well performed and described. While the influence of Mitf on Kcnd3 expression is well supported by evidence, a change in intrinsic properties as a link between Mitf expression and behavioral changes, while plausible, is less convincingly demonstrated. It also has to be noted, that the electrophysiological experiments operate with small sample sizes and the p-values are just below threshold.

Major points:

1) While the study demonstrates a change in isolated A-type currents in M/T cells convincingly, apart from the spontaneous spike rate, there is very little evidence presented for altered active properties in those cells. The probability of spiking upon current injection at different amplitudes in current-clamp configuration, for example, could have been tested, and it is unclear why this, or other more direct measures of excitability were not shown.

Reply: We attempted current injection experiments with 110 pA current pulses only (no other amplitudes) but the cells showed spontaneous spiking even when holding them at hyperpolarized holding potential (-90 mV). This makes any conclusions about the effect of current injection hard to interpret. We therefore decided to remove such experiments out of our current clamp protocol, which already involved various procedures for each cell under both voltage and current clamp, including monitoring the spontaneous spiking activity under current clamp. Our preliminary data suggest that spikes appear faster in the mutant in response to 110 pA current pulses, and the interspike interval is shorter in these in response. However, this needs to be examined further along the lines suggested by the reviewer. In the new Fig 2f we have examples of spikes on an expanded time scale, and these indicate that the duration of the spikes is shorter in the mutant than the WT.

2) In the behavioral dishabituation assay, significant changes are shown in Mitf(mi-vga9/+) heterozygotes. However, there is no mention in the text of any change in the potassium channel expression in heterozygotes. This would be an important piece of information to confirm that the observed behavioral changes are related to Kcnd expression in the olfactory bulb mitral cells.

Reply: We agree that this would be an interesting experiment to do. However, we have found it difficult to perform since Kcnd3 expression is not limited to MT cells in the olfactory bulb so qPCR would not be able to answer the question. Secondly, although the RNAscope method is quantitative to an extent we do not believe we can use it to reliably show intermediate difference in expression without using high number of mice. Newer methods such as Digital Spatial Profiling (NanoStrings), that might be able to generate this data in a reliable fashion, are out of our reach at present. We have therefore not yet been able to answer this question but are actively looking for methods to do this in the near future.

3) The author’s hypothesis regarding ET cells is unclear. While their model is presented for projection neurons in general, ET cells are certainly not following the proposed mechanism in Figure 6. While there is an activity-dependent increase of Mitf in those cells, as well a significant effect of Mitf on Kcnd3 expression, there is no activity-dependent regulation of Kcnd3. This is especially difficult to interpret, as ETCs have a bigger drop in Kcnd3 in Mitf mutants than MTCs.

The paper should present a hypothesis of how the observed effects in ETCs are compatible with the proposed mechanism.

Reply: We realized early that the ETC do not behave in the same way as the Mitral cells, neither with regards to c-Fos nor Kcnd3. This is discussed in several places in the manuscript:

“Mitf is also expressed in a subset of tufted cells, the external tufted cells (ETC), which are excitatory and synapse with MC, but do not project out of the OB (Hayar, Karnup et al. 2004, De Saint Jan, Hirnet et al. 2009, Whitesell, Sorensen et al. 2013). “

” External tufted cells (ETC), a subclass of tufted cells, which drive olfactory output (Hayar, Karnup et al. 2004) and link OSN with M/T neurons (Gire, Franks et al. 2012), do not show increased expression of c-Fos under baseline conditions in the mutant. “

„The expression of both c-Fos and Mitf, but not Kcnd3, was increased upon activity in the ETC. This demonstrates that the MCs and ETCs, which have different functions in the olfactory neuronal circuit, also have a different intrinsic response to activity.” ,

We have therefore focused our work on the key projection neurons: the Mitral cells. It is perhaps not surprising that ETC and Mitral cells behave differently, as they have distinct roles in odorant detection. One plausible explanation would therefore be that as the ETC drives MC activity it is more efficient to regulate a key neuron in a network, rather than two which interact linearly. As we are not able to come up with a hypothesis regarding the regulation of ETC activity, we have put forth a direct and simple hypothesis regarding the key projection neurons of OB, thus excluding the ETCs. The regulation of intrinsic plasticity in different neurons of the OB network will undoubtedly turn out to be complex. We feel that describing these differences between these two neuronal types, does not lessen the importance of our findings. However, the most direct explanation for the different transcriptional behavior of the ETC is the fact that ETC show large compensatory changes in expression of Kcnd2. This is therefore the functional explanation for the reduced effect in the mutant ETC.

We have added the following sentence to the discussion section of the manuscript to underline this:

„One possible interpretation is that the transcription of Kcnd3 is not regulated by Mitf in the ETCs during activity as the adaptive response is more efficient or faster when it occurs only in the key output neurons, rather than also in neurons which drive their activity. It is also noteworthy that ETC show increased expression of Kcnd2 in the mutant (Figure 7), which would dampen the effect of reduced Kcnd3 and could explain reduced effect on ETC´s activity” p. 25

4) The compensatory increase in Kcnd2 presented in Figure 10 is on par, if not stronger than the decrease of Kcnd3 expression in Mitf(mi-vga9/mi-vga9) animals. It is unclear, and the authors should present a reasonable explanation, how this doesn’t prevent the loss of A-type currents in M/T cells.

Reply: Our study shows that Mitf regulates potassium currents in primary M/T neurons of the OB. We also show that in vivo the activity of these neurons is affected. As neuronal activity is highly regulated, there are compensatory mechanisms likely both at the level of the cells (such as increased expression of Kcnd2) and at the networks level i.e. changes in level of inhibition. It is the strength of our study to be able to uncover in the primary cells the effect of the loss of Mitf, without the confounding effects of the network. Another explanation could be that compensatory changes take time and that this occurs more slowly in cell cultures in vitro. It is not feasible to explain why the compensatory mechanisms on transcriptional level do not fully prevent the loss of the A-current, as we are measuring the A-current and observing the reduction. It could be argued that without the increase in kcnd2, the effect on the A-current would be more pronounced. It also can explain why the ETC show a different response.

We have added the following sentence to the results section of the manuscript to clarify this:

„ This increase in the expression of Kcnd2 underlines the tight regulation of neuronal activity. Without this change, the effects on the A-current would likely be larger, especially in the ETC.

p. 21

5) The spike amplitudes displayed on the current clamp traces of Figure 2 are very small: The Results section mentions amplitudes of 54 and 62 mV, while the example traces on Figure 2f displays less than 30 mV spikes measures from baseline in wild type, and barely over 30 mV in mutants as a representative example, which raises the question whether spontaneous spike rates could have been influenced by variability in patch-clamp recording quality. At the displayed time scale, it is difficult to assess spike shape, but the lack of afterhyperpolarization, and the fast time course of EPSPs, taken together with the small spike amplitude, are much more characteristic of granule cells than mitral cells. The authors should display a blown-up version of the recorded traces to address these concerns and allow a better assessment of the spike waveform.

Reply: Unfortunately, there was an error in the voltage calibration of Fig 2f as the calibration bar should indicate 20 mV rather than 10 mV. This has now been corrected. We have also added blown-up examples of spikes from both recorded traces to the figure, to assess the spike waveform. In both the mutant and WT traces there are no afterhyperpolarizations after spikes, while the duration of the individual spikes in the mutant is shorter than in WT.

Minor points:

1) Page 18, last paragraph: Evidence for Kv4 protein expression on the M/T cell plasma membrane at the protein level is available in published literature and could be cited here (e.g. Wang et al. 2015 J Comp Neur and Kollo et al. 2008 Eur J Nsci).

Reply: This has been added.

2) Page 18, last paragraph: How were the cell boundaries defined when counting fluorescent dots?

Reply: As stated in the method section, the distribution of mRNA of the marker gene was used to determine this:

„...by encircling the cloud of Tbr2 dots and using this area to count the fluorescent dots of interest” p. 14

3) Figure 5c: The colors of the plot (blue and red) do not match the colors mentioned in the legend (red and green).

Reply: This has been fixed.

4) Multiple locations in the text: when discussing spontaneously occurring EPSPs, the expression “miniature EPSP” is used. This is generally used for recordings done in TTX, where release is AP independent. “Spontaneous EPSP” would be a more appropriate expression.

Reply: We have altered the text as suggested, replacing miniature with spontaneous on p. 10 and p. 20.

-

Reviewer #2

This manuscript studies the interaction between the neuronal activity of specific neurons in the olfactory bulb and the expression of MITF. The thesis of the manuscript is that the activity of projection neurons in the OB drives the expression of MITF, which in turn regulates the potassium current I¬A through the regulation of the subunits Kcnd3/Kv3.4. This, in turn, affects the sensitivity of the neurons to their synaptic inputs and thus acts as a homeostatic mechanism to regulate the activity of projection neurons of the OB. I find the paper written well and interesting. I am not an expert in some of the methods used by the authors (mainly mFISH and molecular biology), but I find them adequate to support their point by point conclusions. However, while each of the points is well made, there are some significant gaps in the story as a whole. For example:

There is no demonstration of changes in the activity of OB projection neurons in vivo in the mutant mice. I appreciate that the authors wanted a clear demonstration of the effect of the mutation on activity on the specific cells, which is right and nice, but if there are some other mechanisms that change the activity in vivo, then the argument that MITF is the regulator is weaker. So when in figure 5, the author assumes that there are changes in activity that cause the change in behavior, the argument would be much stronger if they would show those changes in the activity.

Similarly, the authors only study the effect of the mutant (i.e., reduction in MITF), but they do not show the effect of elevation in MITF. However, they feel comfortable speculating that an increase in MITF will cause a reduction in activity (pp 22) even though this was not demonstrated in the data at all. For now, as far as I can see, they can only claim one direction of the cycle but not the complete one.

Similarly, the mutant is not targeted only to the OB. So, while there are clear changes in the OB due to the mutation, the authors state that there are other effects (e.g., microphthalmia), so the change in behaviour may result from other effects of MITF in other neuronal systems just as well. In conclusion, I find the study compelling and interesting, but the different levels or experiments are not tight together very strongly. I suggest that the authors will take one of two paths. One, make changes to the text to better reflect the relationship between what they find and what they claim or Two, add more intermediate level experiments such as overexpressing MITF and/or recording activity in the OB in vivo in the mutant and strengthen their support of the claims. Please see more specific comments below.

Reply: We divide our reply to this into three independent sections.

Activity of OB neurons in vivo: We do show that there are changes in the expression of c-fos, which is a community standard for measurements of neuronal activity in vivo. However, it is clear that our in vitro data, while instrumental to being able to draw clear inferences about the role of Mitf, cannot be expected to be fully replicated in vivo for example due to compensatory changes in inhibitory neuronal networks. We acknowledge this and explain in the following manner in the manuscript:

„Importantly, electrophysiological analysis in primary neurons shows that changes in potassium currents are cell-autonomous and not due to changes in OB circuits. Detailed analysis of the electrophysiology of M/T neurons in OB slices and in vivo is needed to further dissect the consequences for olfaction.”

Pleiotropy of the MITF mutation; The reviewer is correct in noting that the lack of Mitf leads to other changes outside the OB, which could possibly affect olfaction. We would like to note that within the OB, Mitf is clearly expressed quite specifically in the M/T cells. These neurons are central to olfaction, and Mitf expression has not been detected in other neuronal system connected with olfaction (our data, Ohba 2015, Allen Brain Atlas). While difficult to rule out, it seems implausible that Mitf is affecting other neuronal systems, given its strong expression in the OB and the defects in Mitf target genes we have uncovered in these same neurons. This concern is however, why our in vitro data from mutant neurons in culture is so valuable. This allows us to focus on the effect in a single cell type and both bypass pleiotropy and complex compensating mechanisms within the OB or in general the olfactory system. This is also the strength of single cell RNA in situ - allowing us to focus on a single cell or population of cells. There are currently no conditional loss of function alleles of Mitf available in the mouse but it will be of great interest to generate them or to examine the effect of conditional loss of Mitf in M/T neuron.

Speculations on increased Mitf expression leading to reduced activity: Our data shows that Mitf is required for the appropriate expression of Kcnd3 and that this connection can occur directly via transcriptional regulation. We also show that Kcnd3 shows increased expression following activity, and that this does not occur in the absence of Mitf. Hence, we have data showing the importance of Mitf for regulation of activity as well as the response to activity. It is of interest that the transcription of Mitf is increased - but this is not a key part of our model. The reason is that Mitf and Kcnd3 expression occurs at the same time, so Mitf transcription can not be the direct cause. Importantly, we therefore do not claim that it is the level of Mitf which regulates the expression of Kcnd3, only that Mitf is required. The actual mechanisms is likely c-Fos binding. Hence, overexpression of Mitf might or might not affect Kcnd3 without activity. We have read the text carefully in order to make sure this is not unclear. Also, we do not consider our hypothesis of Mitf funtion to be a speculation, but rather a model which fits the current data.

We have sought to clarify this in the text in the following manner:

"We propose a hypothesis for a functional role of Mitf in projection neurons of the olfactory bulb, where it regulates the intrinsic excitability of the neurons following activity induction, by reducing the neuronal firing rate of neurons through its regulation of Kcnd3 expression. In this model, the activity of OSNs leads to an increase in Mitf expression, and an Mitf-dependent increase in the transcription of Kcnd3 in the output neurons of the OB. It is possible that it is the presence of MITF, rather than its level or postranslational state, which guides the expression of Kcnd3 following activity. An increased I¬A-current decreases the likelihood of the generation of action potentials and reduces activity leading to decreased sensitivity of the glomeruli in question (Figure 12). Conversely, lack of activity in a glomerulus could lead to the opposite mechanism and increased sensitivity (Tyler, Petzold et al. 2007)"

p. 24

Specific Comments:

R2-1

Figure 2A-D:

The difference between WT and mitf mutant look convincing, but the statistics are really borderline (0.049). Why is that?

Reply: We have looked carefully at the statistical analysis and it is clear that the p-value for the A-type current is 0.049. The reason for that may be discerned from Fig 2d, which shows that the range of the A-type current in the WT cells is wide, while the current in the mutant cells is at the low end, but with one “outlier”. The sample size is 12 cells of each genotype.

R2-2

Figure 2A-D: In both B and C the recordings from the mutant, but not WT, show at least one example of an escape current (un-clamped spike?). This suggests that the VC is compromised by the cell geometry (the current source is away from the recording location). The authors should provide an image or reconstruction of the recorded cell to show that there is no outstanding morphological difference between the WT and mutant (I don’t think there is a need for detailed morphological comparison but just a bold eye comparison).

Reply: We have looked carefully at all the recordings isolating the delayed rectifier current as shown in 2B (the Fig 2C is the difference between A and B and thus any “escape current” or spiking, interference etc in 2B will appear there), in both the mutant and WT cells. We find that in 3 mutant cells out of 12 a brief inward current deflection appears shortly after the -40 mV voltage step, but only in the range of subsequent hyperpolarizing voltage steps greater than -50 mV. If the membrane potential of these 3 cells was stepped in succession from -90 mV to -40 mV and after that to a more hyperpolarizing step, then a brief inward current deflection would appear, resembling an escape current. But that is not the voltage range where the delayed rectifier current is evoked; in that range (above -50 mV) there was never any inward current deflection. The majority of cells did not show this deflection, but we think that the cell shown is still representative of the recordings from mutant cells, and the deflection does not appear during the delayed rectifier current. The measured series resistance of the cells included in this analysis was always within the same range, suggesting that there was no escape current. In addition, cell capacitance was not significantly different between the two genotypes, as indicated in the manuscript (see page 9). This suggests that there is no gross difference in cell morphology between these conditions.

R2-3

The analysis of mEPSPs on pp18 is confusing. First, it looks like that they are not minis and but rather spontaneous (i.e., triggered by presynaptic spikes). Secondly, if the firing frequency of all cells increases, one would expect an increase in the frequency of these EPSPs, which you don’t find. Thirdly, one possible reason for the insignificance you see is because they might trigger APs, and then you don't count them. It looks like this analysis should either be dropped or done on EPSCs (in VC) and not in CC.

Reply: We agree with the reviewers that these are most likely spontaneous EPSPs, and we have replaced the term miniature with spontaneous. The frequency of these is higher in the mutant (as one might expect) but did not reach statistical significance. The possibility raised by the reviewer that a higher frequency may trigger more spikes, which then are not counted as EPSPs, is certainly present. Given these caveats, we have taken the advice of the reviewer and removed the analysis of the frequency of spontaneous EPSPs from the revised manuscript.

R2-4

I have little experience with the behavioural results presented in figure 5D-G. They clearly are significant, but the effects, for an inexperienced eye, seems rather weak.

Reply: The time spent at the novel odorant shows that there is a distinct and significant difference in olfactory behaviour, most likely due to the fact that the mutant mice discern odour better or are more sensitive.

Minor:

1. Supp figures are referenced by regular numbers, which creates confusion. For example, Pp16 - figure 7 is referenced straight after figure 1, and on pp17 figure 2 comes and then figure 9. Can’t you use figure 1, figure s1, figure 2, figure s2 ...

Reply: This has been fixed. All figures have now been renumbered, as eNeuro does not allow supplementary figures.
